# Intelligent Sensing: The Emerging Integration of Machine Learning and Soft Sensors Based on Hydrogels and Ionogels

**DOI:** 10.1002/advs.202517851

**Published:** 2025-11-21

**Authors:** Wenqing He, Rumin Lin, Suixiu Kong, Mengyi Qiang, Lingqi Huang, Bing Dai, Xi Yao, Lei Su, Xueji Zhang

**Affiliations:** ^1^ College of Materials and Energy Guang'an Institute of Technology Guang'an 638000 P. R. China; ^2^ Guangdong Laboratory of Artificial Intelligence and Digital Economy (SZ) Shenzhen University Shenzhen 518060 P. R. China; ^3^ School of Biomedical Engineering Marshall Laboratory of Biomedical Engineering Shenzhen University Medical School Shenzhen University Shenzhen 518060 P. R. China; ^4^ Department of Biomedical Sciences City University of Hong Kong Hong Kong 999077 P. R. China; ^5^ School of Environmental and Natural Resources Zhejiang University of Science and Technology Hangzhou 310023 P. R. China; ^6^ College of Intelligent Textile and Fabric Electronics Zhongyuan University of Technology Zhengzhou 450007 P. R. China

**Keywords:** hydrogels, intelligent sensing, ionogels, machine learning, wearable sensors

## Abstract

Intelligent sensing means the capability of systems to perceive, learn, analyze, and predict based on external stimuli, mimicking the cognitive functions of the human brain. With the assistance of machine learning algorithms for data processing, soft sensors made from hydrogels and ionogels possess intelligent sensing abilities. Here, the recent advances of hydrogel‐ and ionogel‐based soft sensors are comprehensively investigated and summarized, with a specific focus on machine learning‐implemented applications, including handwriting/gesture/object/motion/speech recognition, health monitoring, food detection, and beyond. With current limitations and future perspectives discussed, the fusion of the two is envisioned that can accelerate the development of intelligent sensing in the areas of human‐machine interface (HMI), health care, and soft robotics.

## Graphical abstract

By integrating with machine learning (ML), hydrogel‐ and ionogel‐based soft sensors are gaining human brain‐like capabilities to perceive, learn, and predict. This review summarizes their advances, focusing on ML‐powered applications such as handwriting/gesture/object/motion/speech recognition, health monitoring, food detection, and beyond. This integration is accelerating development in human‐machine interfaces, healthcare, and soft robotics.

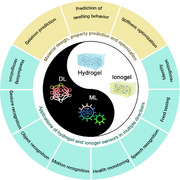

## 1 Introduction

Sensors have been widely used in various applications such as tactile perception,^[^ ^1^ ^]^ human‐machine interaction systems,^[^ ^2^, ^3^ ^]^ and health care,^[^ ^4^ ^]^ transducing external physical, chemical, and biological stimuli into electrical or other measurable signals.^[^ ^5^ ^]^ They are usually required to demonstrate superior biocompatibility and mechanical properties, enabling sustained interaction with uneven surfaces, including skin, muscles, and hair.^[^ ^6^ ^]^ Conventional sensors based on circuit board technology and/or silicon‐based materials exhibit inherent limitations in flexibility and conformability, restricting their applicability in such contexts.^[^ ^7^ ^]^ Thereby, the development of soft sensors that can adapt to geometric nonlinearities and deformations has gained significant attention, offering enhanced adaptability and functionality for complex working conditions.^[^ ^8^, ^9^ ^]^ Progress in this field has been driven largely by the creation of soft composite materials incorporating conductive fillers such as liquid metal,^[^ ^10^, ^11^, ^12^, ^13^ ^]^metal nanowires,^[^ ^14^, ^15^, ^16^, ^17^ ^]^ and metal nanoparticles.^[^ ^18^, ^19^, ^20^ ^]^Among these soft materials, hydrogels and ionogels have been widely used as soft sensors in human‐machine interfaces,^[^ ^21^, ^22^ ^]^ healthcare,^[^ ^23^, ^24^, ^25^ ^]^ soft robotics^[^ ^26^ ^]^ and intelligent systems,^[^ ^27^, ^28^ ^]^ due to their excellent mechanical properties, high sensitivity, and good conductivity.^[^ ^29^, ^30^, ^31^ ^]^ They can adapt to form a compliant contact on various dynamic and uneven surfaces, such as skin, hair, joints, and muscle. As popular materials for sensing matrices, hydrogel and ionogel‐based sensors work in a similar mechanism but with their own advantages. Specifically, hydrogels, with their superior biocompatibility and structural similarity to human tissues, have been recognized as excellent substrate candidates for making wearable sensors, especially in the biomedical fields.^[^ ^32^ ^]^ Ionogels, by incorporating ionic liquids, offer advantages such as freeze resistance and dehydration resistance,^[^ ^33^, ^34^ ^]^ making them especially suitable for sensing under harsh and complex environmental conditions such as high vacuum, high and low temperatures. However, challenges such as low sensing accuracy, insufficient intelligence, and limited versatility of detection capabilities remain unresolved.^[^ ^7^ ^]^ Overall, on the one hand, precise and intelligent sensing requires conformal contact to enable the accurate acquisition of diverse multimodal data.^[^ ^35^, ^36^, ^37^, ^38^ ^]^ On the other hand, advanced data processing techniques are essential to ensure efficient, accurate, and reliable analysis and interpretation of the collected data.^[^ ^39^ ^]^

Machine learning (ML), a subset of artificial intelligence (AI), has emerged as a transformative technology, providing advanced and efficient methods for data processing and prediction. ML focuses on developing algorithms that enable computers to automatically learn from data and improve their performance over time without explicit programming.^[^ ^40^ ^]^ Deep learning (DL) is a specialized subset of ML that excels at processing large and complex datasets using multilayer neural networks by mimicking the human brain's neural network. Both ML and DL have achieved revolutionary progress in pattern recognition,^[^ ^41^ ^]^ intelligent robots^[^ ^42^, ^43^ ^]^ and other tasks. While ML usually requires manual feature extraction and works well with smaller datasets, DL automatically extracts features and typically requires large amounts of data, computational resources, and longer training time. The rapid development of soft sensing technology and the rising demand for personalized healthcare and HMI solutions have made this field highly promising and of great interest. In this context, ML and DL have the potential to overcome the aforementioned challenges by leveraging their respective strengths to improve the reliability and efficiency of soft sensors.

**Figure 1:**
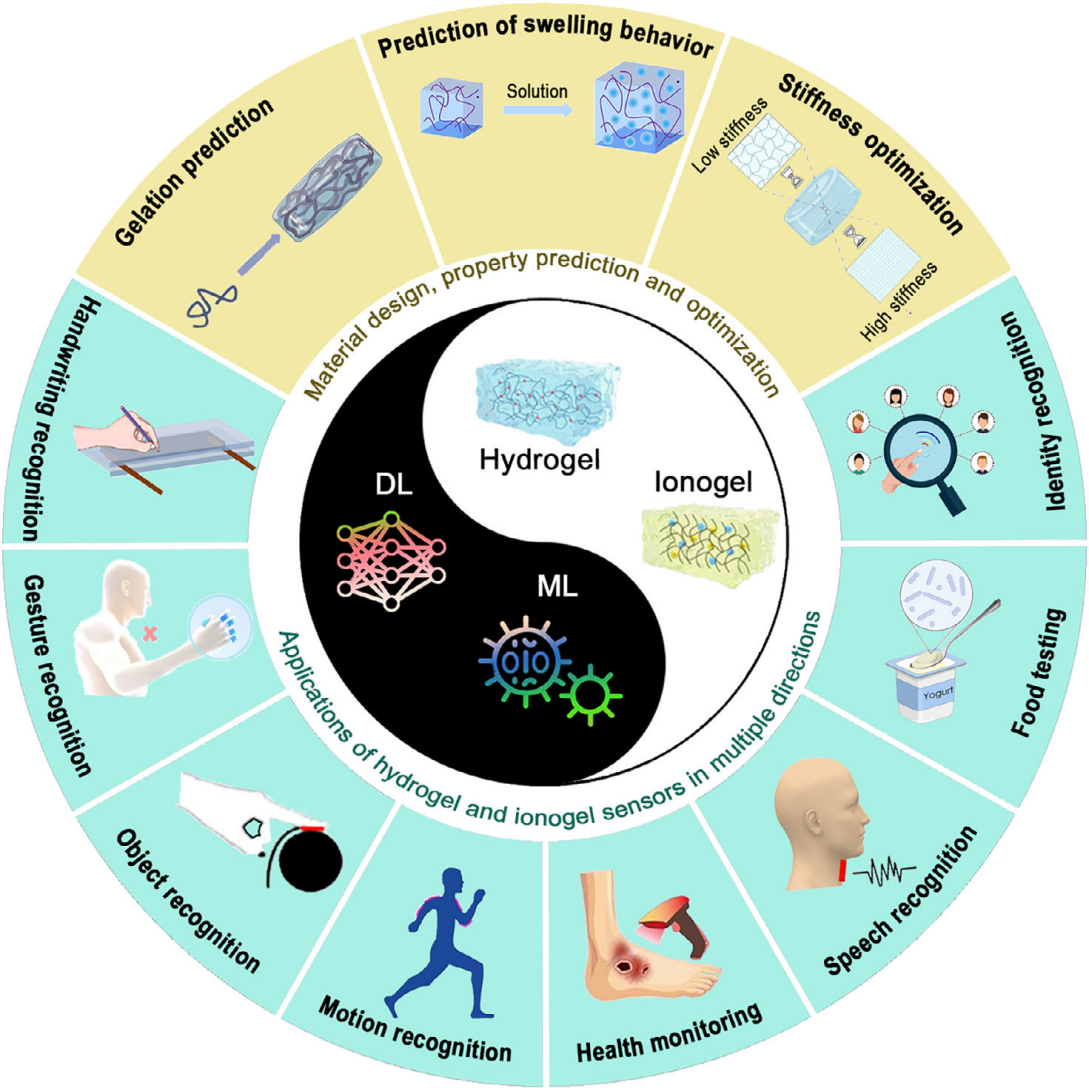
The integration of machine learning and deep learning with hydrogels and ionogels in the material design and optimization. Reproduced with permission.^[^ ^48^ ^]^ Copyright 2023, Elsevier. Reproduced with permission.^[^ ^49^ ^]^ Copyright 2023, American Chemical Society. Reproduced with permission.^[^ ^50^ ^]^ Copyright 2023, American Chemical Society. as well as sensing applications.^[^ ^22^, ^51^, ^52^, ^53^, ^54^, ^55^, ^56^ ^]^ Reproduced with permission.^[^ ^22^ ^]^ Copyright 2023, Elsevier. Reproduced with permission.^[^ ^51^ ^]^ Copyright 2024, Elsevier. Reproduced with permission.^[^ ^52^ ^]^ Copyright 2023, American Chemical Society. Reproduced with permission.^[^ ^53^ ^]^ Copyright 2022, Elsevier. Reproduced with permission.^[^ ^54^ ^]^ Copyright 2023, American Chemical Society. Reproduced with permission.^[^ ^55^ ^]^ Copyright 2022, American Chemical Society. Reproduced with permission.^[^ ^56^ ^]^ Copyright 2024, Wiley.

Recently, some reviews have explored general wearable sensors^[^ ^44^ ^]^ and their development with ML.^[^ ^8^, ^39^, ^45^, ^46^, ^47^ ^]^ However, the design and applications of intelligent sensing, incorporating ML and soft sensors, have not been systematically explored or thoroughly analyzed. This review provides a detailed investigation and discussion of the integration of ML and soft sensors based on hydrogel and ionogel. First, the characteristics and preparation of different hydrogels and ionogels were summarized. Then, the categories and principles of traditional machine learning and deep learning were briefly introduced. We then focus on the role of machine learning algorithms in the design, properties prediction, and optimization of hydrogels (**Figure** **1**). Moreover, we highlight the application of hydrogel and ionogel sensors assisted with ML techniques, including areas such as handwriting/gesture/object/motion/speech/identity recognition, health monitoring, and food testing. With the combination of ML and soft gel sensors, the performance and prediction/perception accuracy can be significantly enhanced in various practical applications. Finally, the advantages and challenges of ML in gel sensor applications were illustrated, unlocking significant potential in material design, health monitoring and management, and human‐machine interaction.

## 2 Basic Concepts of Machine Learning

Machine learning (ML) algorithms differ from traditional data processing methods by automatically learning patterns from data, which enables them to manage complex, high‐dimensional datasets. While conventional approaches rely on predefined rules and manual feature selection, ML algorithms identify features through training data without explicit programming. They can adapt to changing data environments, enhancing their predictive capabilities as more data is introduced. Additionally, ML algorithms effectively handle noise and uncertainty, resulting in more accurate and robust analyses.

**Figure 2:**
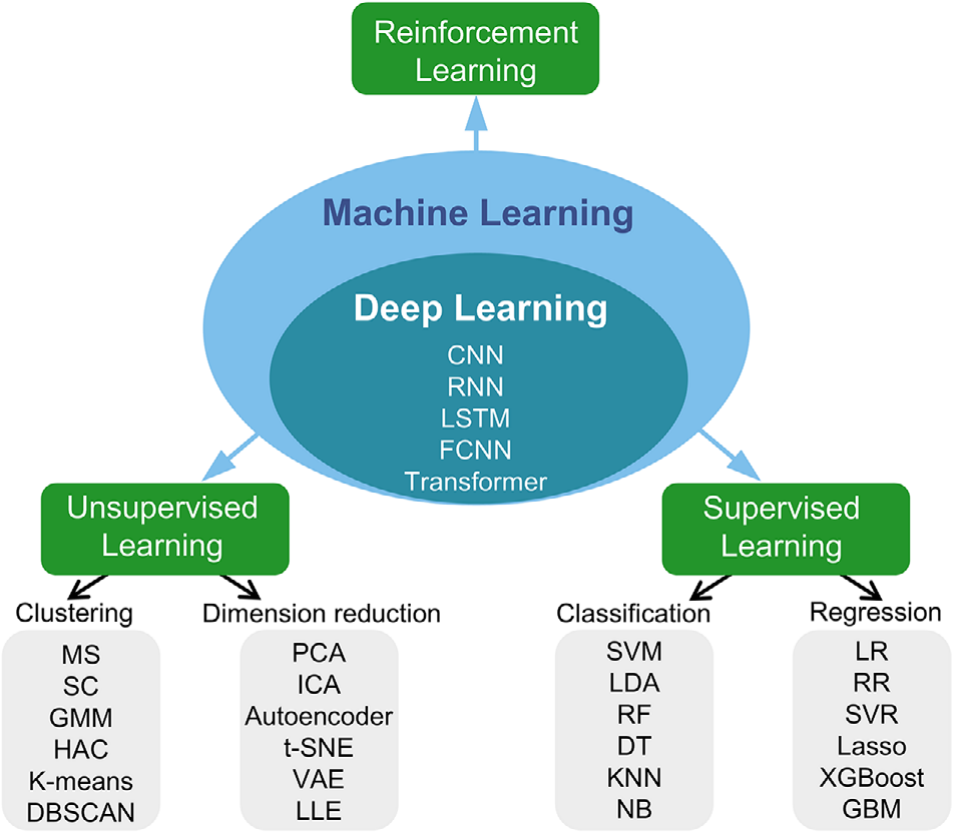
Classification of machine learning techniques. Machine learning is divided into supervised, unsupervised, and reinforcement learning. Deep learning is a subset of machine learning, with common models including convolutional neural networks (CNN), recurrent neural networks (RNN), long short‐term memory (LSTM), fully connected neural networks (FCNN), and transformer models. Supervised learning is categorized into classification and regression, with classification algorithms such as support vector machines (SVM), random forest (RF), and decision trees (DT), k‐nearest neighbors (KNN), naive bayes (NB), while regression algorithms include linear regression (LR), ridge regression (RR), support vector regression (SVR), lasso regression, extreme gradient boosting (XGBoost) and gradient boosting machine (GBM). Unsupervised learning encompasses clustering and dimensionality reduction techniques, with clustering algorithms like mean shift (MS), spectral clustering (SC), Gaussian mixture models (GMM), hierarchical agglomerative clustering (HAC), k‐means, DBSCAN, and dimensionality reduction methods including principal component analysis (PCA), independent component analysis (ICA), autoencoders, t‐distributed stochastic neighbor embedding (t‐SNE), variational autoencoder (VAE), and locally linear embedding (LLE).

**Figure 3:**
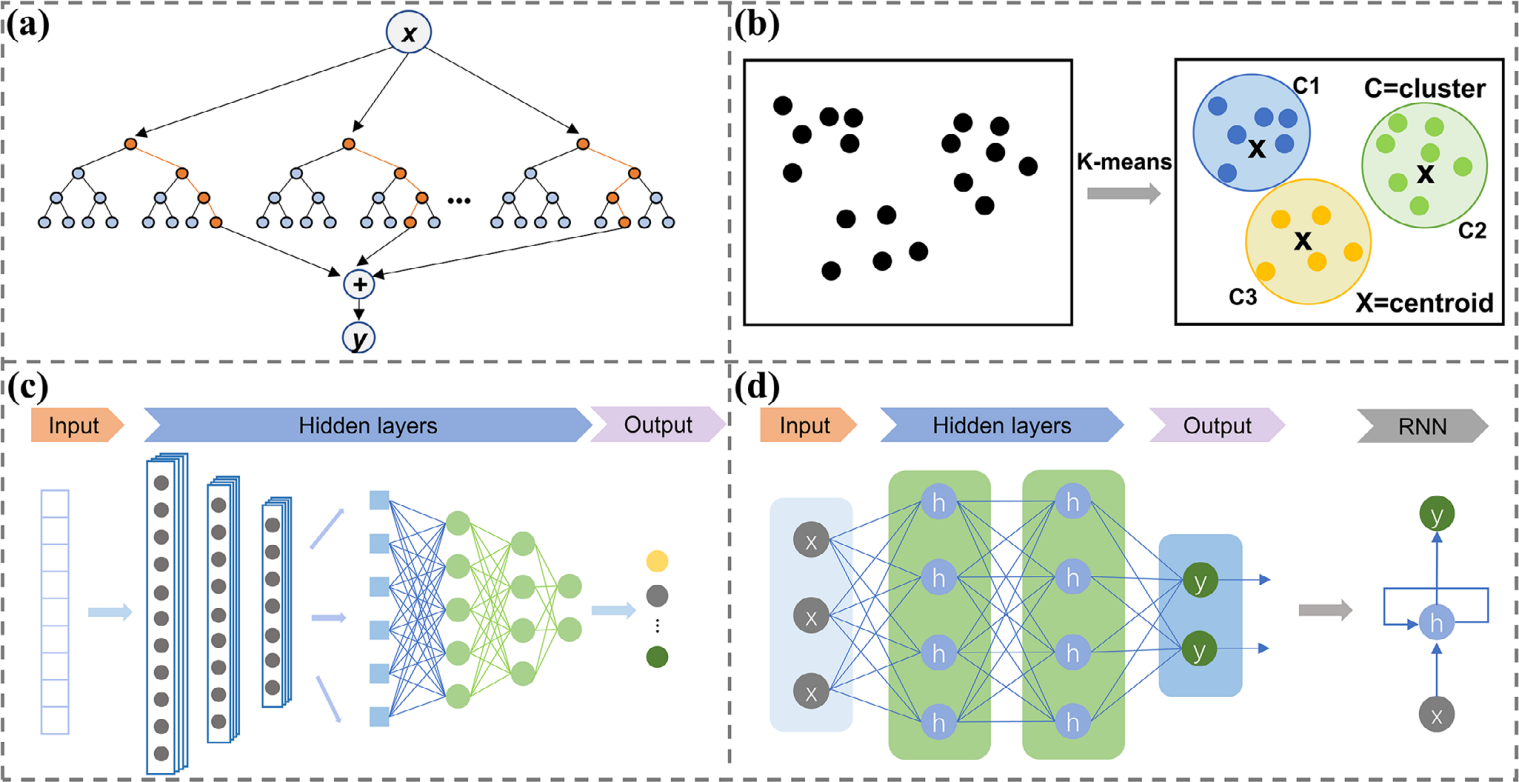
The working principle of machine learning: a) A decision tree performs classification or regression through a series of decision nodes (orange) and leaf nodes (blue). Each node represents a feature or condition, and the leaf nodes represent the final classification or prediction result. b) The k‐means algorithm assigns data points to different clusters (C1, C2, C3), each with a centroid (X representing the centroid). The algorithm continuously adjusts the position of the centroids to minimize the within‐cluster distance variance. c) The feature extraction processes the input layer of the convolutional neural network (CNN) through multiple convolution and pooling layers. Then the classification results are generated through a fully connected layer. d) A recurrent neural network (RNN) unit specifically includes the input, hidden, and output layers. The recurrent mechanism of the RNN shows the input data (x) enters the hidden state (h), subsequently, the hidden state generates the output (y) and feeds back into itself, forming a loop.

Machine learning algorithms are commonly categorized into supervised, unsupervised, and reinforcement learning (**Figure** **2**).^[^ ^57^ ^]^ Supervised learning uses labeled data to model input‐output relationships, enabling predictions for new data. Key algorithms include linear regression, support vector machines, and tree‐based methods, each having unique advantages. Linear regression predicts continuous variables by fitting a line that minimizes the difference between predicted and observed values, based on the assumption of a linear relationship.^[^ ^58^ ^]^ It is simple, computationally efficient, and easily interpretable, but is restricted to linear relationships, making it ineffective for modeling complex, non‐linear data. Support Vector Machine (SVM) is a versatile algorithm for classification and regression tasks.^[^ ^59^ ^]^ It identifies the optimal hyperplane in the feature space to separate different classes. SVM performs well on high‐dimensional and small‐sample datasets and handles non‐linear problems effectively through kernel functions. However, it has limitations, including sensitivity to parameter and kernel selection, high computational cost for large datasets, and limited interpretability. SVM is widely used in fields like text classification, image recognition, financial forecasting, and anomaly detection.^[^ ^60^, ^61^, ^62^, ^63^ ^]^ Tree‐based algorithms include decision trees, random forests, and XGBoost. Decision trees split data into regions,^[^ ^64^ ^]^ making them intuitive for tasks like medical diagnosis, customer segmentation, and risk assessment.^[^ ^65^, ^66^, ^67^ ^]^ Random forests improve accuracy and handle complex relationships by combining multiple decision trees (**Figure** **3**a),^[^ ^68^ ^]^ and are applied in feature selection, anomaly detection, and market analysis.^[^ ^69^, ^70^, ^71^ ^]^ XGBoost builds trees iteratively, optimizing performance with parallel computing and regularization, excelling in text classification, network security, and diagnostics.^[^ ^72^, ^73^, ^74^ ^]^ These algorithms are powerful but prone to overfitting, sensitive to outliers, and may face computational challenges with large datasets.

In contrast, unsupervised learning algorithms work with unlabeled data to uncover hidden patterns or structures. These algorithms analyze the data's intrinsic properties without predefined labels. Common types comprise clustering algorithms and dimensionality reduction techniques. Clustering algorithms group data into categories based on similarity. K‐means and DBSCAN are widely used clustering methods. K‐means iteratively assigns data points to K clusters, minimizing squared error within each cluster (Figure 3b).^[^ ^75^ ^]^ It is simple and computationally efficient, but requires predefined clusters (K) and is sensitive to outliers and cluster shapes. K‐means is used in image processing, text clustering, and object detection.^[^ ^76^, ^77^, ^78^ ^]^ DBSCAN, a density‐based method, defines clusters by identifying high‐density regions,^[^ ^79^ ^]^ making it better for irregular clusters and noisy data. It does not require predefined clusters or the handling of noisy data, but may struggle with datasets having varying densities. Dimensionality reduction algorithms reduce the number of features in a dataset while retaining essential information. Key methods include principal component analysis (PCA) and independent component analysis (ICA). PCA projects data onto a new coordinate system using linear transformation,^[^ ^80^ ^]^ maximizing variance and simplifying the dataset. It is computationally efficient and easy to interpret, but only captures linear relationships, limiting its effectiveness for non‐linear data. PCA is widely used in data analysis, image processing, and feature extraction.^[^ ^81^, ^82^, ^83^ ^]^ ICA decomposes data into independent components,^[^ ^84^ ^]^ making it valuable in signal processing.^[^ ^85^ ^]^ It can uncover non‐linear and independent hidden factors, but is computationally complex and depends on specific assumptions about the data.

As data complexity grows, deep learning has become a central approach in machine learning, leveraging multilayer neural networks (deep neural networks) for data processing and feature extraction. Deep learning excels at automatically identifying high‐level features and complex patterns, especially in high‐dimensional data. It has made significant strides in image recognition and natural language processing, performing well in both supervised and unsupervised tasks. Key models include convolutional neural networks (CNN), recurrent neural networks (RNN), long short‐term memory networks (LSTM), residual networks (ResNet), autoencoders, and generative adversarial networks (GAN).

CNNs are central to deep learning, excelling in computer vision and natural language processing.^[^ ^86^ ^]^ They primarily process 2D images but can be adapted for 1D and multidimensional data, making them versatile for various applications. 1D‐CNNs use 1D convolutional kernels to analyze 1D data (Figure 3c), focusing on local patterns and trends. They are effective in time series prediction (e.g., atrial fibrillation detection, traffic flow forecasting)^[^ ^87^, ^88^ ^]^ and signal recognition tasks such as arrhythmia detection and fault detection in bearings.^[^ ^89^, ^90^ ^]^ 1D‐CNNs have also shown remarkable potential in soft‐sensor signal analysis, as most hydrogel‐ and ionogel‐based sensors generate 1D data (e.g., resistance, voltage, capacitance, strain). By extracting local temporal features directly from raw signals, 1D‐CNNs enable accurate gesture and motion recognition without extensive manual feature design. Additionally, the convolution–pooling structure of 1D‐CNNs effectively suppresses noise and improves signal robustness, which is particularly beneficial for flexible sensors that are susceptible to drift and mechanical hysteresis. 2D‐CNNs, the most common type, are designed for 2D data, extracting spatial features from images (e.g., edges, textures) through successive convolutions. They are widely used in medical image classification,^[^ ^91^ ^]^ segmentation,^[^ ^92^ ^]^ and facial recognition.^[^ ^93^ ^]^ 3D‐CNNs handle 3D data, capturing features across both spatial and temporal dimensions. They are used in human action recognition and object recognition tasks.^[^ ^94^, ^95^ ^]^

RNNs, designed for processing sequential data,^[^ ^96^ ^]^ allow the output to depend on the current input and previous states, enabling them to handle temporal dependencies (Figure 3d). They are highly useful for tasks such as natural language processing (NLP),^[^ ^97^ ^]^ speech recognition, and time series forecasting. However, they encounter issues with vanishing and exploding gradients in long sequences. LSTM networks address this by using a gating mechanism (forget, input, output gates) to capture long‐term dependencies and improve sequence coherence.^[^ ^98^ ^]^ LSTMs have shown success in long‐text processing, machine translation, speech synthesis, health applications,^[^ ^99^, ^100^, ^101^ ^]^ and sentiment analysis (**Table** **1**).^[^ ^102^, ^103^ ^]^

**Table 1:** Advantages and disadvantages of different ML algorithms.

Algorithm	Advantages	Disadvantages
Principal component analysis (PCA)	Reduces dimensionality and noise, facilitates feature extraction	Only handles linear relationships; difficult to capture complex features
k‐nearest neighbors (KNN)	Intuitive algorithm, no training time is required	Sensitive to distance metric and k‐value, computationally taxing
Naive bayes (NB)	Simple structure, good with text data	Fails with correlated features
Support vector machine (SVM)	Works in high dimensions, high accuracy, and speed with small datasets, with less influence of outliers	Sensitive to kernel selection, poor performance on large datasets
Decision tree (DT)	Clear logic, interpretable results, can handle irrelevant features, and nonlinear associations.	Prone to overfitting, sensitive to small changes
Logistic regression (LR)	Interpretable & explainable, applicable for multi‐class predictions.	Weak with non‐linear boundaries
Random forest (RF)	Reduces overfitting risk, robust, suitable for large datasets	Requires longer training, less suitable for real‐time classification or regression tasks
Convolutional neural network (CNN)	Strong feature extraction ability, translation invariant, highly parallelizable	Poor with sequential data and long‐term dependencies
Recurrent neural network (RNN)	learns temporal dependencies, simpler than LSTM or Transformer	Vanishing gradient problem
Long short‐term memory (LSTM)	Captures long‐term dependencies, prevents gradient vanishing	Long training time, relatively high complexity
Transformer	Captures global dependencies, fully parallelizable, suitable for multi‐modal dynamic inputs	Requires high computational resources, underperforms LSTM in some dynamical systems.

**Table** **2** summarizes both advantages and disadvantages of the reviewed machine learning algorithms. Other deep learning algorithms have not been explored in depth, and readers are encouraged to explore related resources on their own.^[^ ^104^, ^105^ ^]^ In the following sections, we will explore how machine learning facilitates the design, performance prediction, and optimization of hydrogel materials, as well as introduce the research progress on the multi‐domain applications of hydrogels and ionogels combined with machine learning. These discussions will cover the application of ML technologies in improving the efficiency of hydrogel design, accurately predicting performance, and optimizing material properties. Additionally, we will showcase how machine learning is advancing the latest applications of hydrogels and ionogels in fields such as healthcare, intelligent perception, and industry.

**Table 2:** Material design, property prediction, and performance optimization of hydrogel assisted by machine learning.

Target	Data	ML methods	Results	Refs.
Prediction of dipeptide gelation properties	Structurally diverse hydrogel library comprising 2304 compounds by a combinational approach	Random forest (RF), logistic regression (LR), gradient boosting	Gradient boosting performs best	[139]
Prediction of hydrogel‐formation ability	71 reported nucleotide derivatives	Extreme gradient boosting (XGBoost), RF, decision tree (DT), and LR	LR performs best	[140]
Prediction of tetrapeptide hydrogels	20^4^ cases of Tetrapeptides	Coarse‐grained molecular dynamics (CGMD), ML‐trained regression model, SVM classification	87.1% accurate in predicting hydrogel formation	[141]
Optimize Young's Modulus and gelation time via concentration	Experimental synthesis dataset for GelMA	Artificial neural network (ANN)	Predicted vs. experimental values show strong agreement (R^2^ > 0.97)	[49]
Predict the fracture behavior	Generated simulation data using finite element method (FEM)	PredNet deep learning model	n.a.	[143]
Predict the swelling states	Literature, synthesis and swelling test setup parameters of PNIPAAm	ANN	relative prediction error of 0.11	[144]
Optimization design of hydrogel‐based NHE metamaterials	Initial dataset is constructed by FEM simulation	Back‐propagation neural networks (BPNN), multi‐population genetic algorithms (MPGA)	3D metamaterial achieves ≈82% NHE ratio	[48]
Optimization of strain sensitivity, elongation, fracture energy, hysteresis, and resistivity	Dual‐network hydrogels based on acrylamide (AM) and alginate	BO; RF	Predicted and experimental property trends align	[145]
Cost and time‐efficient optimization	Synthesized ≈1000 photodegradable hydrogels	Droplet microarray for high‐throughput screening, Gaussian process regression models (GPR), and BO	BO identifies hydrogels with increased intensity and lifetime	[146]
Link precursor properties to protein release behavior	Synthesized 126 hydrazone‐cross‐linked in situ‐gelling hydrogels	Automated high‐throughput robotic system, partial least squares (PLS) regression	Predict hydrogel recipes for improved protein release	[147]
Design of PF127/PF68/MK4M‐based thermosensitive hydrogels	Generated experimental data	FormRules v4.03, artificial neural network	Thermosensitive enemas for rapid gelation and protein delivery	[142]

## 3 Soft Sensor Based on Hydrogel and Ionogel

A sensor transforms physical, chemical, or biological parameters into comprehensible and interpretable output signals.^[^ ^106^ ^]^ Hydrogels and ionogels exhibit superior biocompatibility, mechanical properties, and flexibility.^[^ ^107^ ^]^ These characteristics have led to the widespread application of hydrogels and ionogels in wearable and implantable sensors.^[^ ^108^ ^]^ In recent years, these sensors based on hydrogels or ionogels have undergone rapid development across various fields, including human‐computer interaction^[^ ^109^, ^110^ ^]^ and medical diagnosis.^[^ ^111^ ^]^ In this section, we will compare the key characteristics and explore the preparation methods of hydrogel and ionogel, two prominent materials used as soft sensing substrates.

### 3.1 Fundamentals of Hydrogel and Ionogel

A gel is a flexible material consisting of a 3D polymer network that is crosslinked either physically (i.e., with non‐covalent bonds such as hydrogen bonds or ionic bonds) or chemically (i.e., with covalent bonds).^[^ ^112^ ^]^ Gels are classified based on the solvent in which they swell: hydrogels utilize water as the solvent; organogels incorporate organic solvents; ionogels are formed with ionic liquids (ILs) as the solvent; and aerogels are defined by the presence of air as the solvent.^[^ ^113^ ^]^ Among them, hydrogels can absorb and retain large amounts of water, exhibiting a soft and moist gel state similar to that of biological tissues. Hydrogels have experienced significant development since the 1960s. In contrast, ionogels are in the relatively nascent stage.^[^ ^114^ ^]^ Ionogels integrate the characteristics of solid‐state electrolytes and hydrogels, i.e., ionogels not only inherit the flexibility and biocompatibility of hydrogels but also exhibit enhanced electrical conductivity due to the incorporation of ionic liquids.

**Figure 4:**
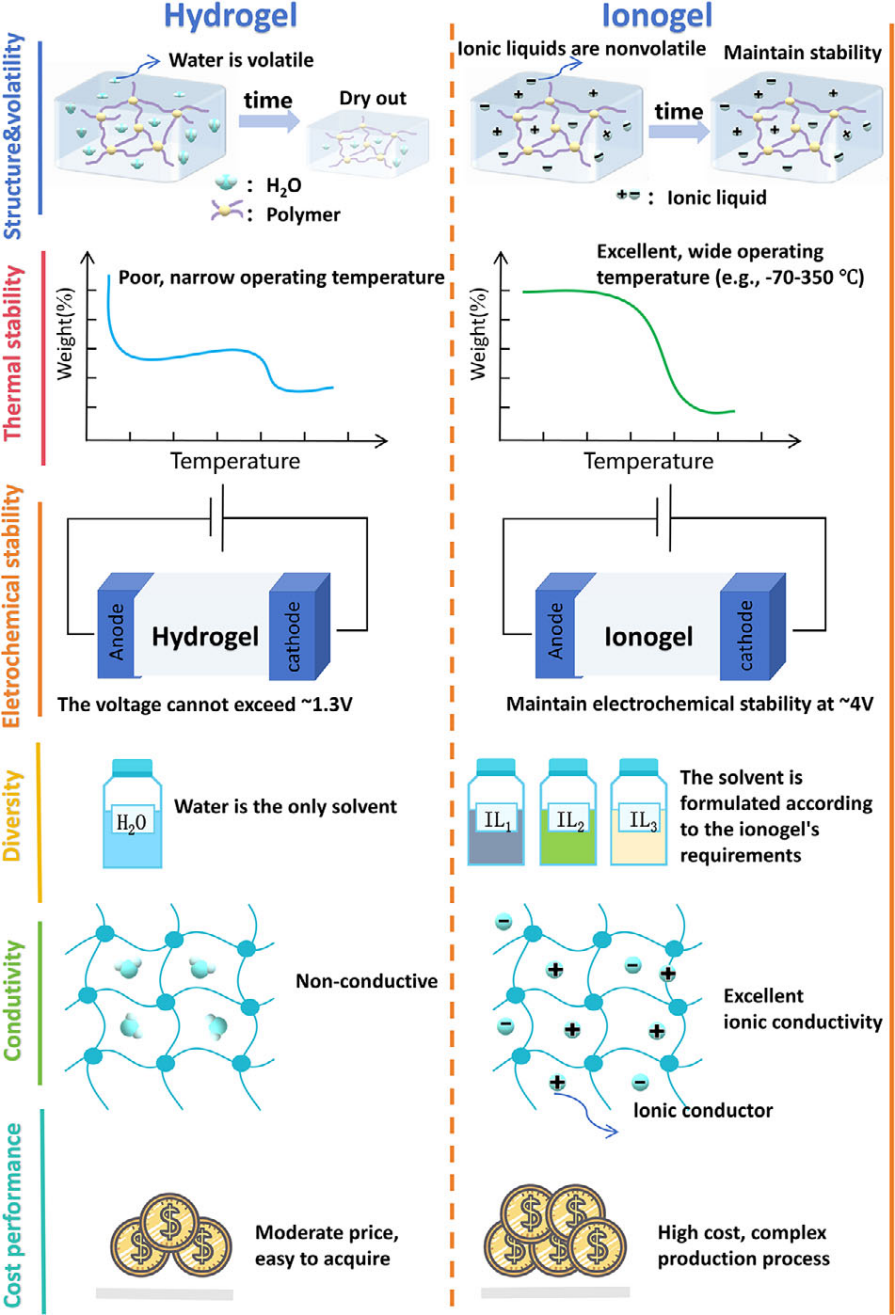
A schematic diagram compares hydrogels and ionogels in terms of structure, volatility, thermal stability, electrochemical stability, solvent diversity, electrical conductivity, and cost performance. Ionogels offer advantages in volatility, thermal stability, electrochemical stability, diversity, and conductivity, whereas hydrogels are more cost‐effective.

A detailed comparison of characteristics between hydrogels and ionogels is shown in **Figure** **4**. First, due to the volatility of different solvents, the structural stability between hydrogels and ionogels is different. Hydrogel will shrink with time due to the evaporation of water, especially in environments with high temperatures and low humidity.^[^ ^114^ ^]^ However, the dimensions of ionogels remain stable over time because of the non‐volatility of ionic liquids. Second, ionogels have better thermal stability than hydrogels. The operational temperature range of hydrogels extends from 0 to 100 °C, corresponding to the freezing and boiling points of water. In contrast, ionogels can be safely utilized within a temperature range of −70 to 350 °C, allowing them to function in extreme environments.^[^ ^115^ ^]^ Third, ionogels usually have better electrochemical stability than hydrogels. Electrolysis of water happens when the voltage exceeds 1.3 V, while ionogels maintain electrochemical stability up to 4 V. Fourth, hydrogels only use water as a solvent, while about 10^18^. Ionic liquids with different anions and cations can be used as solvents for ionogels. Fifth, ionogels exhibit superior conductivity compared to hydrogels. Ionogels have conductive ions, resulting in enhanced ionic conductivity. Last but not least, due to the higher cost of ionic liquids compared to water, hydrogels are generally more affordable and easily available.

### 3.2 Preparation of Hydrogel and Ionogel

Hydrogels are 3D hydrophilic networks that are crosslinked either physically or chemically.^[^ ^116^ ^]^ Physical crosslinking refers to the formation of crosslinking points between molecules through noncovalent interactions (intermolecular interactions), such as ionic bonds and hydrogen bonds. The intermolecular interaction forces in hydrogels produced through physical crosslinking are generally weaker than those of chemical bonds, enabling them to exhibit reversible responses to environmental changes,^[^ ^117^ ^]^ which makes them suitable for the preparation of self‐healing hydrogels.^[^ ^118^ ^]^ Additionally, the synthesis of hydrogels using physical crosslinking methods avoids the use of organic solvents and small‐molecule crosslinking agents and eliminates the presence of toxic covalent crosslinking molecules, thereby possessing advantages such as good biocompatibility and biodegradability.^[^ ^119^ ^]^ However, due to the relatively weak interactions, these hydrogels commonly suffer from issues such as poor mechanical strength, insufficient stability, and a relatively short service time.^[^ ^120^ ^]^

Chemical crosslinking method refers to the process of linking small molecules into polymer chains through chemical reactions of monomers, initiators, and crosslinking agents, resulting in a robust hydrogel network and exhibiting enhanced stability in comparison with physically crosslinked hydrogels.^[^ ^116^, ^119^, ^121^ ^]^ By adjusting the content and ratio of crosslinking agents, the properties of the hydrogels, including their swelling behavior, biodegradability, and mechanical strength, can be altered.^[^ ^116^, ^120^, ^122^ ^]^ However, during the process of chemical crosslinking, toxic reactions may occur during the multiple steps of preparation and purification, making the selection of crosslinking agents crucial.^[^ ^120^, ^123^ ^]^ To possess good biocompatibility, green chemicals or natural crosslinking agents are currently widely used in chemical crosslinking.^[^ ^124^ ^]^

The integration of electrical conductivity transforms hydrogels into conductive hydrogels, paving the way for innovative applications in sensing fields. Conductive hydrogels can be divided into two main categories: ionic conductive and electrically conductive hydrogels. Electrically conductive hydrogels usually utilize carbon materials,^[^ ^125^, ^126^ ^]^ MXenes^[^ ^21^, ^127^ ^]^ and liquid metals (LMs)^[^ ^128^, ^129^ ^]^ as conductive additives. Carbon materials, such as graphite, graphene, graphene oxide (GO),^[^ ^125^, ^130^ ^]^ carbon nanotubes (CNTs), activated carbon, carbon fibers,^[^ ^126^ ^]^ and carbon nanoparticles, were optimal conductive additives for the electrical hydrogel preparation due to their superior electrical conductivity and cost‐effectiveness. MXenes are a family of 2D transition metal carbides, nitrides, or carbon nitrides, obtained by selective etching of the MAX phase, exhibiting large surface area, high electrical conductivity, and intrinsic flexibility. LMs have emerged as ideal candidates for fabricating high‐performance conductive hydrogels due to their high conductivity, fluidic properties, and low toxicity. As for ionic conductive hydrogels, the swelled aqueous phase provides numerous channels for ion migration, thereby demonstrating excellent electrical conductivity. Compared with electrically conductive hydrogels, ionic conductive hydrogels possess transparency. The ionic conductive hydrogels primarily include Li^+^, Fe^3+^, K^+^,^[^ ^131^ ^]^ Al^3+^,^[^ ^51^ ^]^ Ca^2+^,^[^ ^28^ ^]^ and other ions. The primary challenge of ionic conductive hydrogels is their insufficient conductivity. To improve the conductivity, multiple ions were incorporated within the hydrogel matrix.^[^ ^100^ ^]^ Furthermore, the introduction of both conductive fillers and ions improved the hydrogel's electrical properties, enabling a combination of electronic and ionic conductivity.^[^ ^132^ ^]^

Ionogels are usually prepared based on hydrogels by introducing ionic liquids (ILs) into the synthesis process. This can be achieved by methods such as swelling polymer networks with ILs, in situ polymerization, or solvent exchange.^[^ ^113^ ^]^ The simplest method for preparing ionogels is directly mixing ILs with a solid polymer network, which confines the ILs within the polymer matrix.^[^ ^133^ ^]^ The affinity between polymers and ILs significantly affects the composition of the ionogel due to the polymer network's maximum swelling capability.^[^ ^134^ ^]^ In situ polymerization or gelation of monomers in ILs enables the preparation of ionogels with transparency and stability via photo‐induced or thermal‐induced polymerization reaction.^[^ ^135^ ^]^ However, polymerization or gelation is sometimes severely hindered by the presence of ILs. Moreover, thermally induced polymerization requires relatively high temperatures (70–80 °C) and longer reaction times (2–24 h).^[^ ^136^ ^]^ The solvent exchange method is employed when monomer molecules are difficult to dissolve in ILs to form a homogeneous precursor for ionogel preparation,^[^ ^137^ ^]^ The water is swelled in a prepared hydrogel is replaced by ILs, allowing the ionogel preparation with difficult gelation reactions in ILs.^[^ ^120^ ^]^

## 4 Material Design, Property Prediction, and Performance Optimization Assisted by Machine Learning

Hydrogel materials, due to their unique physical and chemical properties, hold extensive application potential in fields such as biomedicine, flexible electronics, and intelligent sensing.^[^ ^128^, ^138^ ^]^In recent years, with advancements in machine learning technology, its application in the design, performance prediction, and optimization of hydrogel materials has become a major research focus. Machine learning can aid in optimizing hydrogel synthesis processes and formulation design, guiding the development of novel functional hydrogels and accelerating the material development process. Moreover, through machine learning models, researchers can extract key features from large experimental datasets, establish relationships between material structure and performance, and efficiently predict critical properties of hydrogels, such as mechanical properties, thermal stability, and conductivity. This integration significantly enhances the accuracy and reliability of hydrogel material design, enabling performance optimization and broadening its practical applications.

In the development of biomaterials, peptide hydrogels have attracted substantial attention due to their unique properties, excellent biocompatibility, and low immunogenicity. However, traditional hydrogel design methods frequently depend on experimental screening and serendipitous discovery, which are inefficient. Li et al. established a structurally diverse hydrogel library comprising 2304 compounds by a combinational approach to address this challenge.^[^ ^139^ ^]^ The relationship between chemical features and dipeptide gelation properties was investigated with machine learning algorithms (random forest, logistic regression, gradient boosting). The results indicated that the gradient boosting algorithm demonstrated the best predictive performance among the tested models. Besides, a dataset of 71 reported nucleotide derivatives was transformed into feature matrices using molecular descriptors to predict the hydrogel‐formation ability of nucleotide derivatives.^[^ ^140^ ^]^ Following feature selection and hyperparameter optimization, four machine learning algorithms, including extreme gradient boosting (XGBoost), random forest (RF), decision tree (DT), and logistic regression (LR), were adopted, with LR performing the most favorable results. The study validated the model's effectiveness by experiment and identified two cation‐independent hydrogels with potential applications, thereby highlighting the role of machine learning in predicting nucleoside‐based hydrogels.

**Figure 5:**
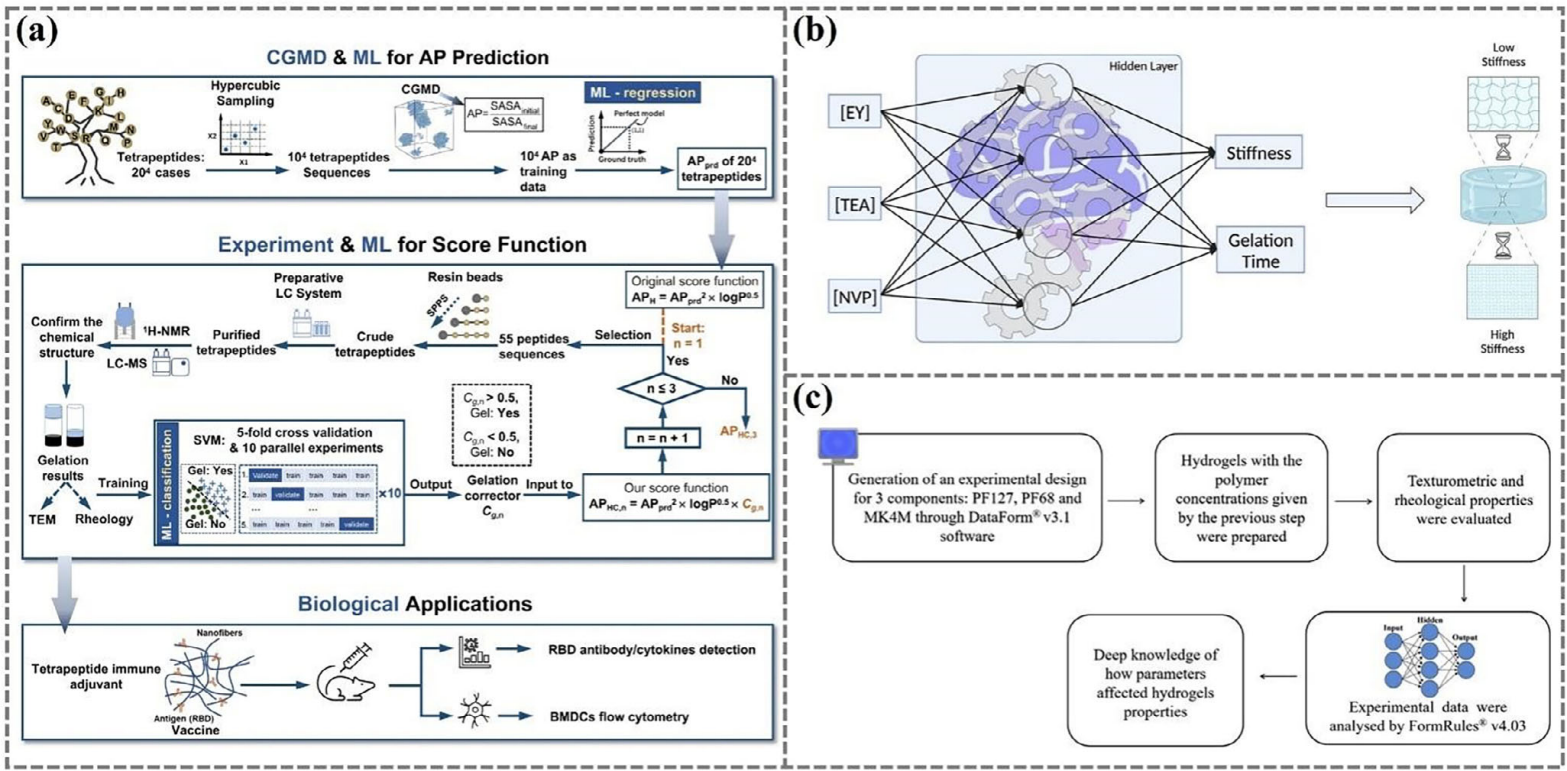
The machine learning‐assisted discovery of tetrapeptide hydrogels, the prediction of hydrogel stiffness and gelation time, and the optimization of hydrogel properties. a) Flowchart of ML‐assisted tetrapeptide hydrogels preparation, including three steps: CGMD and ML for aggregation propensity (AP) prediction, experiment, and ML for score function, and biological applications. Reproduced with permission.^[^ ^141^ ^]^ Copyright 2023, Springer Nature. b) The ANN model, with EY, TEA, and NVP as the input variables, accurately predicts the properties of GelMA hydrogels (Young's modulus and gelation time), enabling the synthesis of hydrogels with customized stiffness. Reproduced with permission.^[^ ^49^ ^]^ Copyright 2023, American Chemical Society. c) Overall chart flow of how AI was introduced to design thermosensitive hydrogels based on three components: PF127, PF68, and MK4M. DataForm v3.1 software, FormRules v4.03 combined with ANN, were incorporated to explore the influence of different parameters on the properties of hydrogels. Reproduced with permission.^[^ ^142^ ^]^ Copyright 2019, Elsevier.

An integrated approach combining coarse‐grained molecular dynamics (CGMD),^[^ ^141^ ^]^ machine learning, and experimental methods was adopted to accelerate the discovery and prediction of tetrapeptide hydrogels (**Figure** **5**a). The CGMD and ML‐trained regression model were utilized to estimate the aggregation propensity (AP). Then, the researchers selected and chemically synthesized 55 peptides for gelation validation based on the available score function AP_H_. The resulting gelation feasibility was used to train the SVM classification model, generating a gelation corrector Cg. An updated function was derived from the input. The entire process above was repeated three times, resulting in 100 out of 165 peptides capable of assembling into hydrogels. Subsequently, the research team generated an 8000‐peptide library, achieving an accuracy of 87.1% in predicting hydrogel formation. Moreover, the designed tetrapeptide hydrogels functioned as immune adjuvants to enhance the response upon the receptor‐binding domain of the SARS‐CoV‐2 virus in a mouse model. This integration of experiment and ML boosts the discovery and application of peptide hydrogels.

In recent years, machine learning algorithms have significantly improved the efficiency and accuracy of hydrogel property prediction and optimization. The prediction and optimization of biomaterials with desired mechanical properties to match the corresponding tissues are crucial for various biomedical applications. Karaoglu et al. established an experimental synthesis dataset for Gelatin methacryloyl (GelMA) hydrogels by varying the concentrations of Eosin Y (EY), triethanolamine (TEA), and N‐vinyl‐2‐pyrrolidone (NVP) (Figure 5b).^[^ ^49^ ^]^ An artificial neural network (ANN) model was developed to optimize the effects of different concentration combinations on the stiffness (Young's Modulus) and gelation time. The study thoroughly examined the correlation between feed concentrations and the resultant stiffness and gelation time, thereby facilitating the optimization of GelMA hydrogels to achieve optimal mechanical properties that better meet the demands of the in vivo microenvironment. The mechanical properties of hydrogels determine how they respond to applied stress, particularly in terms of fracture behavior. Wang et al. utilized the finite element method (FEM) to generate simulation data of hydrogel fracture and developed a PredNet deep learning model to predict the fracture behavior of hydrogels under various loadings with high accuracy.^[^ ^143^ ^]^

Swelling behavior and negative hydration expansion properties are important physicochemical properties of the hydrogel. These characteristics determine the volume changes and phase transition behaviors of hydrogels under varying environmental conditions, which are critical for their performance in practical applications. Researchers used an artificial neural network model to predict the swelling states and characteristics of temperature‐responsive hydrogels (PNIPAAm) by inputting their synthesis and swelling test setup parameters.^[^ ^144^ ^]^ In another study, an optimization design method was developed that integrates back‐propagation neural networks (BPNN) with multi‐population genetic algorithms (MPGA) specifically for designing hydrogel‐based metamaterials with negative hydration expansion (NHE) effect. The designed 3D metamaterials achieved an NHE ratio approaching the theoretical limit, reaching ≈82%.^[^ ^48^ ^]^

The machine learning‐assisted optimizations mentioned above primarily focus on one or two specific properties of hydrogels. A Bayesian optimization algorithm was adopted to investigate five key properties of dual‐network hydrogels based on acrylamide (AM) and alginate: strain sensitivity, elongation, fracture energy, hysteresis, and resistivity.^[^ ^145^ ^]^ Through the integration of machine learning, the experimental parameters were precisely adjusted, resulting in hydrogels with enhanced strain sensitivity, elongation, and fracture energy, making them more effective for flexible sensor applications.

The integration of machine learning and a high‐throughput screening platform not only reduces experimental time and material consumption but also efficiently discovers material combinations with superior performance, paving the way for advanced hydrogel performance optimization. Seifermann et al. demonstrated that the combination of high‐throughput screening and machine learning models can optimize the properties of photodegradable hydrogels in a cost and time‐efficient manner.^[^ ^146^ ^]^ Droplet microarray was employed as a miniaturized high‐throughput experimental platform to synthesize ≈1000 hydrogels with different binary compositions. Two multitask Gaussian process regression models (GPR) and Bayesian optimization (BO) were utilized to further optimize the experimental conditions for obtaining useful properties, such as high initial intensity and desired lifetime for 3D cell culture. In another study, researchers synthesized 126 hydrogels using an automated high‐throughput robotic system and conducted a comprehensive characterization, including their mechanical properties, swelling behavior, degradation, transparency, and protein (ovalbumin) release kinetics.^[^ ^147^ ^]^ A partial least squares (PLS) regression model was employed to correlate the input data—such as the concentration, molecular weight, and degree of functionalization of precursor polymers—with the protein release behavior to establish a dynamic model. This approach provides crucial technical support for the rapid identification and optimization of injectable hydrogels related to protein delivery. Garcia‐del Rio et al. developed an AI‐based method to obtain thermosensitive hydrogels suitable for protein rectal administration in inflammatory bowel disease (IBD) (Figure 5c).^[^ ^142^ ^]^ The research team used DataForm v3.01 software to generate an experimental design that included three components: PF127, PF68, and MK4M. Hydrogels with different compositions were prepared, and their texturometric and rheological properties, such as syringeability, bioadhesion, gelation temperature, and viscosity, were subsequently characterized. The generated experimental data were analyzed using FormRules v4.03 and input into an artificial neural network to predict the impact of each component on hydrogel characteristics. By optimizing these parameters, the study aimed to produce easily manageable and highly bioadhesive enemas capable of undergoing rapid sol‐gel transitions at body temperature, facilitating rectal protein delivery.

Current research in machine learning applied to gel materials focuses on the design and performance optimization of hydrogels (Table 2). Studies primarily utilize easily modularized and synthesized hydrogels, such as peptide‐based protein hydrogels and in situ gelling hydrogels. These materials enable high‐throughput sample preparation and testing, which in turn accelerates the design and optimization cycle. In the future, the design and optimization of complex hydrogels and ionogels will advance rapidly, driven by progress in AI technologies such as machine learning.

## 5 Application of Soft Sensors Based on Hydrogel and Ionogel Assisted by Machine Learning

### 5.1 Handwriting Recognition

Handwriting recognition is an important application of hydrogel sensors. Hydrogel sensors, with their high sensitivity and flexibility, can capture subtle movements and pressure changes during the handwriting process, offering new possibilities for high‐precision handwriting signal recognition once integrated with machine learning. This integration plays a critical role in text recognition. Handwriting recognition applications are mainly divided into two categories: one is the direct recognition of handwriting signals on hydrogel material, and the other is recognizing handwriting information by monitoring the movement of fingers or hands, without a specific writing interface.

#### 5.1.1 Recognition of Writing Signals on Hydrogel

Hydrogel materials have emerged as an innovative medium in handwriting recognition due to their softness, deformability, and high sensitivity to external stimuli. In these applications, a user can write directly on a surface coated with hydrogel. The hydrogel's high sensitivity enables it to capture subtle mechanical changes, thereby generating precise electrical signals, including resistance, voltage, and current. These signals can then be analyzed using machine learning algorithms to accurately recognize written content.

**Figure 6:**
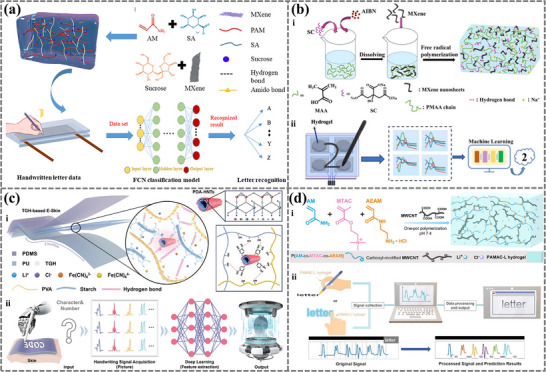
The application of machine learning‐assisted hydrogel sensors in handwriting recognition. (a) The fabrication of PGMS hydrogel and its application as a sensor for handwritten letter recognition by FCN algorithm with resistance signals collected. Reproduced with permission.^[^ ^50^ ^]^ Copyright 2023, American Chemical Society. b i) The preparation of PMAA@MXene hydrogel. (b ii) PMAA@MXene hydrogel sensing matrix was used to collect current data for handwriting recognition assisted by machine learning. Reproduced with permission.^[^ ^151^ ^]^ Copyright 2024, American Chemical Society.c i) Sandwich structure of TGH‐based e‐skin consisted of PDMS layer, PU layer, and TGH functional layer. Reproduced with permission.^[^ ^153^ ^]^ Copyright 2024, Wiley. c ii) Flow chart of signature recognition and biometric authentication powered by deep learning. d i) One‐pot polymerization process to synthesize the nanocomposite hydrogel PAMAC‐L hydrogel. d ii) Handwriting recognition process by attaching the PAMAC‐L hydrogel on fingers and training with machine learning. Reproduced with perm^[^ ^138^ ^] ^Copyright 2022, Elsevier.

Based on the relative resistance changes caused by the pressure applied by the writer during the writing process on hydrogel materials, recent research has incorporated MXene and poly(3,4‐ethylenedioxythiophene) (PEDOT) into hydrogels to create a composite double‐network (DN) hydrogel that can capture resistance change signals generated during writing.^[^ ^148^ ^]^ Integrating these signals with a 1D convolutional neural network (1D‐CNN) method has achieved an accuracy of 94% in recognizing Arabic numerals. Based on the 1D‐CNN algorithm, Zhang et al. introduced tannic acid (TA) and MXene into the polyacrylamide (PAM)/ carboxymethyl chitosan (CMC) double‐network hydrogel to develop a multifunctional conductive hydrogel sensor, which showed an accuracy of 98% for Arabic numeral recognition.^[^ ^149^ ^]^ Additionally, a self‐adhesive, anti‐freezing PGMS hydrogel was made from an aqueous solution of acrylamide (AM), sodium alginate (SA), sucrose, and MXene with N,N′‐methylenebisacrylamide (MBAA) as the crosslinker and 2,2′‐azobis[2‐(2‐imidazolin‐2‐yl) propane] dihydrochloride (AIBI) as the thermal initiator (**Figure** **6**a).^[^ ^50^ ^]^ The resistance signals generated during the writing process were collected and further trained with the fully convolutional network (FCN) algorithm. A handwriting recognition accuracy of 98.1% for English letters was obtained. MXene/polypyrrole/hydroxyethyl cellulose (MXene/PPy/HEC) strain sensor demonstrated reliable sensing performance, including long‐term durability and fast response. By using machine learning, different Arabic numerals, Chinese characters, and English letters were recognized with an accuracy higher than 96%.^[^ ^150^ ^]^ The variations in current signals generated during the writing process on hydrogels can also be adopted for handwriting recognition. Wang et al. fabricated PMAA@MXene hydrogels with MXene as a conductive filler and trisodium citrate dihydrate (SC) as a cross‐linking agent (Figure 6b i).^[^ ^151^ ^]^ The four obtained hydrogels were placed between two 2 × 2 matrix circuit boards (Figure 6b ii) to capture current signals. Various machine learning models, including support vector machine (SVM), logistic regression (LR), decision tree (DT), random forest (RF), k‐nearest neighbors (KNN), 1D‐CNN, and recurrent neural network (RNN), were used to evaluate the handwriting recognition performance. Ultimately, the optimal model of RNN achieved a recognition accuracy of 97.44%.

Voltage signal changes induced by the pressure during the writing process can also be utilized for hydrogel sensors. A type of triboelectric nanogenerator, PHM‐TENG, which employs polyacrylamide (PAM)/hydroxypropyl methylcellulose (HPMC)/MXene hydrogels as the functional electrodes, has been assembled with VHB tapes as triboelectric and encapsulation layers.^[^ ^152^ ^]^ PHM‐TENG can generate different voltage signals corresponding to various handwriting letters. Different neural networks were employed to process and analyze these voltage signals. The 1D‐CNN model demonstrated the best recognition performance, achieving an accuracy of 97%.

Additionally, variations in handwriting habits among different users can be accurately identified using deep learning algorithms, enabling precise recognition of individual handwriting styles.^[^ ^125^, ^126^, ^129^, ^153^ ^]^ Li et al. fabricated thermogalvanic hydrogel (TGH) via a one‐pot method with polydopamine‐halloysite nanotubes (PDA‐HNTs), polyvinyl alcohol (PVA), starch, LiCl, K_4_[Fe(CN)_6_], and K_3_[Fe(CN)_6_]·3H_2_O (Figure 6c i), resulting in TGH with freezing tolerance and non‐drying abilities due to Li^+^(H_2_O)n hydration structure formation.^[^ ^153^ ^]^ The self‐powered electronic skin (e‐skin) comprised a PDMS film as the writing layer, a PU film as the packaging layer, and the TGH as the functional layer. Writing directly on the e‐skin produced corresponding electrical signals by integrating sensing and thermoelectric conversion, which were subsequently extracted and trained using the ResNet‐34 algorithm (Figure 6c‐ii). The system achieved an average recognition accuracy of 98.46% for letters and 96.23% for digits. Furthermore, the e‐skin can distinguish signals generated by different users writing the same content, enabling effective signature recognition and identity verification, with an accuracy of 92.97% when validating six distinct individuals.

#### 5.1.2 Handwriting Recognition Based on Monitoring Finger Movement

Another approach for handwriting recognition lies in integrating hydrogel materials into wearable devices, such as gloves, finger sleeves, and e‐skin on fingers. These devices effectively capture the motion trajectories of fingers. Hydrogel sensors can record signals related to the speed, direction, and force of finger movements in real‐time. These data are then transmitted to machine learning models, which facilitate the recognition of written characters or symbols.^[^ ^129^, ^138^, ^154^, ^155^ ^]^ For example, Wu et al. synthesized the nanocomposite hydrogel PAMAC‐L through a facile one‐pot free radical polymerization of acrylamide (AM),^[^ ^138^ ^]^ 2‐methacryloyloxy ethyl trimethylammonium chloride (MTAC), and 2‐aminoethyl acrylamide hydrochloride (AEAM) in the presence of carboxyl‐modified MWCNT and LiCl (Figure 6d i). The PAMAC‐L hydrogel was employed to monitor forefinger joint motions, and a custom software was developed to collect and process signals (Figure 6d ii). With the help of machine learning, PAMAC‐L successfully recognized complex human behaviors, such as handwriting in the air and on paper, and translated them into digital text (e.g., “letter” and “hydrogel”) with high accuracy.

A patterned LM layer was sandwiched between two P(AAm‐co‐AAc)/Zr^4+^ hydrogel layers to assemble a piezoresistive strain sensor. Strain signals of the fingers during the handwriting process were collected and analyzed by machine learning, further exhibiting the ability to recognize handwritten words.^[^ ^155^ ^]^ A notable advantage of wearing hydrogel sensors on the hand for finger movement monitoring during writing is that there is no need for a physical writing surface. It makes these sensors particularly suitable for applications in virtual reality, showing tremendous potential for future human‐machine interaction.

### 5.2 Gesture Recognition

Hydrogel and ionogel sensors possess high conductivity, low interfacial impedance, excellent adhesion, and biocompatibility, enabling them to monitor subtle hand movements that cause changes in relative resistance, voltage, and electromyography (EMG) signals.^[^ ^156^ ^]^ In modern gesture recognition technology, by integrating these sensors with machine learning algorithms, the sensors can detect and process the signal changes caused by hand movements, classify and predict these signals, thus achieving efficient gesture recognition. This section will discuss the application and research progress of hydrogel and ionogel sensors combined with machine learning in gesture recognition by focusing on three key categories: relative resistance, voltage changes, and EMG signals.

#### 5.2.1 Relative Resistance Signals

Relative resistance change is a commonly used sensing principle in gesture recognition, particularly in flexible wearable devices. These sensors monitor the changes in electrical resistance caused by finger or joint movements to identify gestures. Hydrogels and ionogels, with their high sensitivity and deformability, can capture the minute resistance changes during hand movements. These changes are then translated into gesture information, allowing the system to recognize various hand motions and gestures.

**Figure 7:**
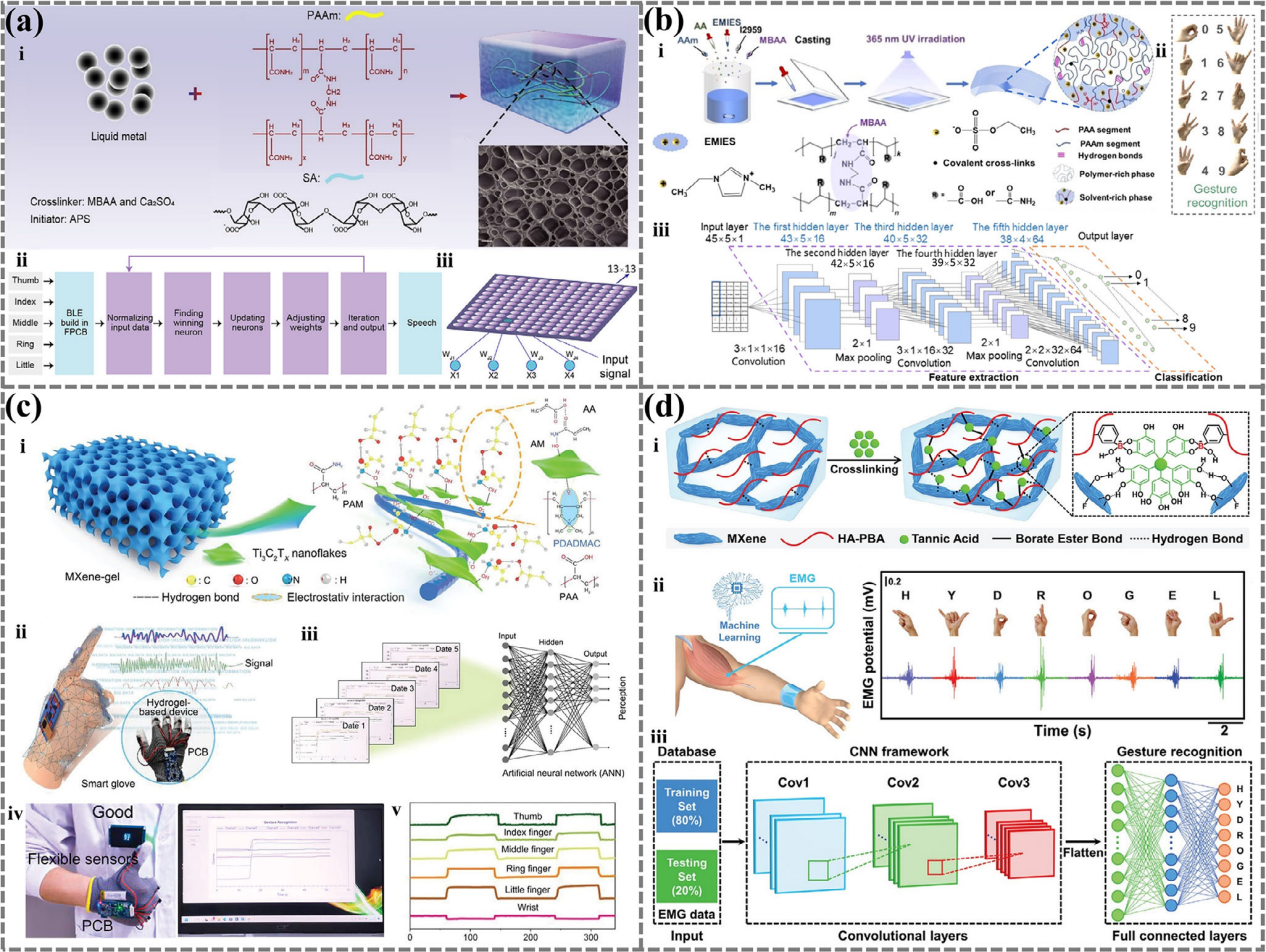
The application of machine learning‐assisted hydrogel and ionogel sensors in gesture recognition. a i) Liquid metal (LM) particles were encapsulated into sodium alginate (SA) and polyacrylamide (PAAm) networks to form the LM‐H with a double network structure. Reproduced with permission.^[^ ^128^ ^]^ Copyright 2023, Wiley. Signal processing method a ii) of the gesture recognition based on LM‐H sensors assisted by machine learning a iii). b i) Schematic of the preparation process of the copolymer ionogel by copolymerizing acrylic acid (AA) and acrylamide (AAm) with 1‐ethyl‐3‐methylimidazolium ethyl sulfate (EMIES, ionic liquid) under UV irradiation. Reproduced with permission.^[^ ^160^ ^]^ Copyright 2024, Wiley.b ii) Numbers (0–9) and their corresponding gestures for gesture recognition. b iii) The process of gesture recognition using a deep convolutional neural network (DCNN), comprising three convolutional layers, two maximum‐pooling layers, and one fully connected layer. c i) A schematic diagram of the structure of MXene‐gel. Reproduced with permission.^[^ ^127^ ^]^ Copyright 2023, Wiley. (c‐ii) Schematic diagram of the MXene‐gel‐based wearable device for sign language‐to‐Chinese character conversion. (c‐iii) An artificial neural network (ANN) consists of input, hidden, and output layers. c iv) The wearable conversion system translates the sign language gesture into the Chinese character "good" and transmits it to the display system. c v) Voltage changes of each finger and wrist during gesturing. d i) The preparation of the bioadhesive hydrogel (MXene/HA‐PBA/TA). Reproduced with permission.^[^ ^21^ ^]^ Copyright 2024, Wiley. d ii) The electromyography (EMG) signals of eight different sign language gestures based on the hydrogel sensors. d iii) The workflow of a convolutional neural network (CNN) used for sign language gesture recognition.

Numerous studies have utilized hydrogel sensors to capture the subtle movements in sign language, converting the relative resistance change signals generated by these motions into text or speech through machine learning algorithms.^[^ ^28^, ^131^, ^157^ ^]^ This technology is significant in enabling communication between deaf‐mute individuals and normal people. Ma et al. developed a stretchable liquid metal‐embedded hydrogel (LM‐H) by encapsulating liquid metal (LM) particles into a PAAm‐SA hydrogel network composed of polyacrylamide and sodium alginate (**Figure** **7**a i).^[^ ^128^ ^]^ LM‐H‐based sensors can detect and collect the relative resistance changes caused by finger movements at different bending angles, process the signals, and wirelessly transmit them to a customized mobile phone (Figure 7a ii). By integrating a self‐organizing map (SOM) (Figure 7a iii), the system accurately recognizes several gestures and translates them into speech with a response time of 0.21 s. This provides an ideal strategy for real‐time communication between deaf‐mute individuals and others.

Graphene was added to a hydrogel for conductivity improvement, except for the liquid metal. PSTG (PAM/SA/TG) hydrogel was made through thermal polymerization of acrylamide (AM), sodium alginate (SA), and tannic acid‐reduced graphene oxide (TA‐rGO, TG). With the 1D‐CNN algorithm, PSTG sensors achieved a 100% recognition accuracy rate for nine distinct types of gestures.^[^ ^158^ ^]^ Monitoring finger movements with this wearable recognition system can promote patients’ engagement in rehabilitation training. PCG hydrogel composed of polyacrylamide (PAM), carboxymethyl cellulose (CMC), and reduced graphene oxide (rGO) was reported to work as a strain, pressure sensor.^[^ ^159^ ^]^ Combined with 1D‐CNN and a customized data acquisition system, PCG hydrogel can be used as a one‐handed wearable keyboard to capture relative resistance changes for gesture recognition with an accuracy of 98.13%, advancing the accessibility for one‐armed or disabled users.

Conductive OSA‐(Gelatin/PAM)‐Ca Hydrogel with superior mechanical properties, self‐healing, and adhesive capabilities was designed by bridging gelatin and PAM network with oxidized sodium alginate (OSA) through Schiff base reaction and adding conductive calcium chloride.^[^ ^28^ ^]^ The hydrogel‐based sensor precisely recognized 10 gestures both before and after self‐healing with three machine learning algorithms, i.e., KNN, decision tree, and SVM. Based on mutual interaction between water molecules and silk fibroin (SF), MXene, Ca^2+^, and H^+^, Duan et al. made a breakthrough at the material level by developing a reversible water‐modulated biomimetic hyper‐attribute‐gel (Hygel) e‐skin with multiple sensing capabilities,^[^ ^27^ ^]^ including pressure, strain, humidity, and temperature sensing, along with excellent physical‐chemical properties (weak acidity, temperature adaptivity, fire‐retardant ability) and skin‐like reconfigurability. Assisted with a 1D‐CNN model, Hygel e‐skin achieved dynamic gesture recognition and intuitive virtual control of in‐game actions.

Compared to hydrogels, ionogels possess ionic conductivity and thermal stability, enabling them to respond sensitively to resistance changes caused by hand movements without interference from environmental temperature. Sun et al. synthesized a copolymer ionogel using acrylamide (AAm)/ acrylic acid (AA) as monomers and 1‐ethyl‐3‐methylimidazolium ethyl sulfate (EMIES) as the solvent (Figure 7b i).^[^ ^160^ ^]^ They combined the ionogel sensor with a deep convolutional neural network (DCNN) (Figure 7b ii) to build a dynamic gesture recognition system. This system comprehensively analyzes the relative changes in resistance and temporal sequence information from wearable sensors placed on five fingers, accurately classifying 10 distinct gestures from five participants with an accuracy of 93.66% (Figure 7b iii). Moreover, it eliminates the influence of specific sensors or individual characteristics (e.g., device update, action angles, and movement speeds). Zhang et al. developed organic ionic gel (POIG) by dissolving PVA into a mixed solvent of ethylene glycol (EG) and ionic liquid (1‐butyl‐3‐methylimidazolium tetrafluoroborate) and subsequently freeze‐induced crystallization.^[^ ^161^ ^]^ This POIG exhibited remarkable mechanical strength, flexibility, and energy dissipation abilities, advancing protective equipment and wearable sensors. Supported by a CNN algorithm, a POIG‐based wearable sleeve can detect relative resistance changes caused by finger flexion and achieve gesture recognition.

#### 5.2.2 Voltage Signals

Voltage change is another employed parameter for gesture recognition. Sensors track voltage changes caused by finger or hand movements to identify gestures. Zhao et al. synthesized the P(AA‐co‐AM)/MXene@PDADMAC semi‐interpenetrating network (semi‐IPN) hydrogel through template copolymerization of acrylic acid (AA) and acrylamide (AM) in the presence of MXene@PDADMAC (Poly(diallyldimethylammonium chloride)) (Figure 7c i), demonstrating excellent flexibility, high conductivity, and water retention.^[^ ^127^ ^]^ The proposed wearable translation system (Figure 7c ii) included a sign language interpretation module and a Chinese character display component (Figure 7c iv). Movements of the fingers and wrist induced the hydrogel to stretch or squeeze, altering resistance and resulting in changes in voltage division across each channel (Figure 7c v). Various gestures were identified, converted, and displayed on the screen with an artificial neural network (ANN) algorithm (Figure 7c iii), enabling daily communication between the deaf and normal individuals. A kind of PAA/PVA‐based conductive organohydrogel was designed utilizing bayberry tannin as a crosslinker, possessing superior physical, self‐healing, adhesive, and anti‐freezing properties.^[^ ^51^ ^]^ The organohydrogel‐based smart glove can monitor multi‐angle free movement of the hand and interpret the sign language by analyzing collected voltage data with the MLSTM‐FCN model.

#### 5.2.3 Gesture Recognition Based on Electromyographic (EMG) Signals

EMG signals are commonly used bioelectrical signals that measure the potential changes generated during muscle contractions, enabling effective gesture recognition. This method directly reflects muscle activity and is particularly well‐suited for detecting subtle muscle movements. EMG‐based gesture recognition is frequently applied in areas such as prosthetic control, rehabilitation training, and ergonomics. Compared to gesture recognition methods based on resistance or voltage changes, EMG provides more direct information about muscle movements, making it suitable for recognizing more complex gestures and actions.

The Mxene hydrogel (Mxene/HA‐PBA/TA) was made using phenylboronic acid grafted hyaluronic acid (HA‐PBA), tannic acid (TA), and MXene (Ti_3_C_2_T_x_) nanosheets assembled within a polymer network, exhibiting improved electrical conductivity and superior sensing capabilities (Figure 7d i).^[^ ^21^ ^]^ These hydrogel sensors were applied to the volunteers’ right arm to monitor electromyogram (EMG) signals generated by eight distinct gestures (Figure 7d ii). This gesture recognition system, integrated with a CNN algorithm (Figure 7d iii) facilitated barrier‐free communication with hearing‐impaired individuals. Moreover, HA‐PBA/PVA/MXene hydrogels were prepared for EMG detection and gesture identification.^[^ ^162^ ^]^ These Mxene hydrogels demonstrated multifunctionalities, such as adhesive, healable, photothermal, and EMI shielding properties. Another conductive hydrogel (PSDM) was fabricated via a solution‐gel process using PVA and silk fibroin (SF) as gel networks, dopamine‐modified polypyrrole (DAPPy), and MXene as conductive additives, realizing a sensitive test of ECG and EMG signals.^[^ ^163^ ^]^ The collected EMG signals were analyzed by XGBoost learning model for gesture recognition with high accuracy.

Unlike the EMG single‐mode sensors for gesture recognition, an epidermal electrode and a pressure sensor, composed of NaCl‐TA‐PAM hydrogel and Foam‐PAM hydrogel, respectively, were developed and integrated to collect EMG and forcemyography (FMG) signals simultaneously.^[^ ^164^ ^]^ After analysis by machine learning, the results indicated that combining EMG and FMG signals significantly improved recognition accuracy compared to using EMG signals alone.

### 5.3 Object Recognition

#### 5.3.1 Resistance to Pressure and Capacitance to Pressure

When objects come into contact with sensors based on hydrogels and ionogels, they can detect subtle pressure variations and convert them into analyzable electrical signals for object recognition. By integrating machine learning algorithms, these pressure signals can be processed to accurately identify different objects based on their unique pressure distributions. This approach is particularly useful in applications where tactile feedback is essential, such as in robotic grippers or touch‐sensitive interfaces. Based on Ti_3_C_2_T_x_ MXene/lithium salt (LS)/poly(acrylamide) (PAM)/poly(vinyl alcohol) (PVA) hydrogel, a study developed a stretchable and conductive MXene‐based organohydrogel (M‐OH) using a simple immersion strategy in a water/glycerol binary solvent.^[^ ^165^ ^]^ This M‐OH sensor exhibited high pressure sensitivity, capable of detecting resistance variation under pressure as low as 12 Pa. Additionally, a pixelated M‐OH sensor array facilitated pressure mapping of different objects, achieving an object recognition accuracy of 97.54% with the assistance of a DNN algorithm.

**Figure 8:**
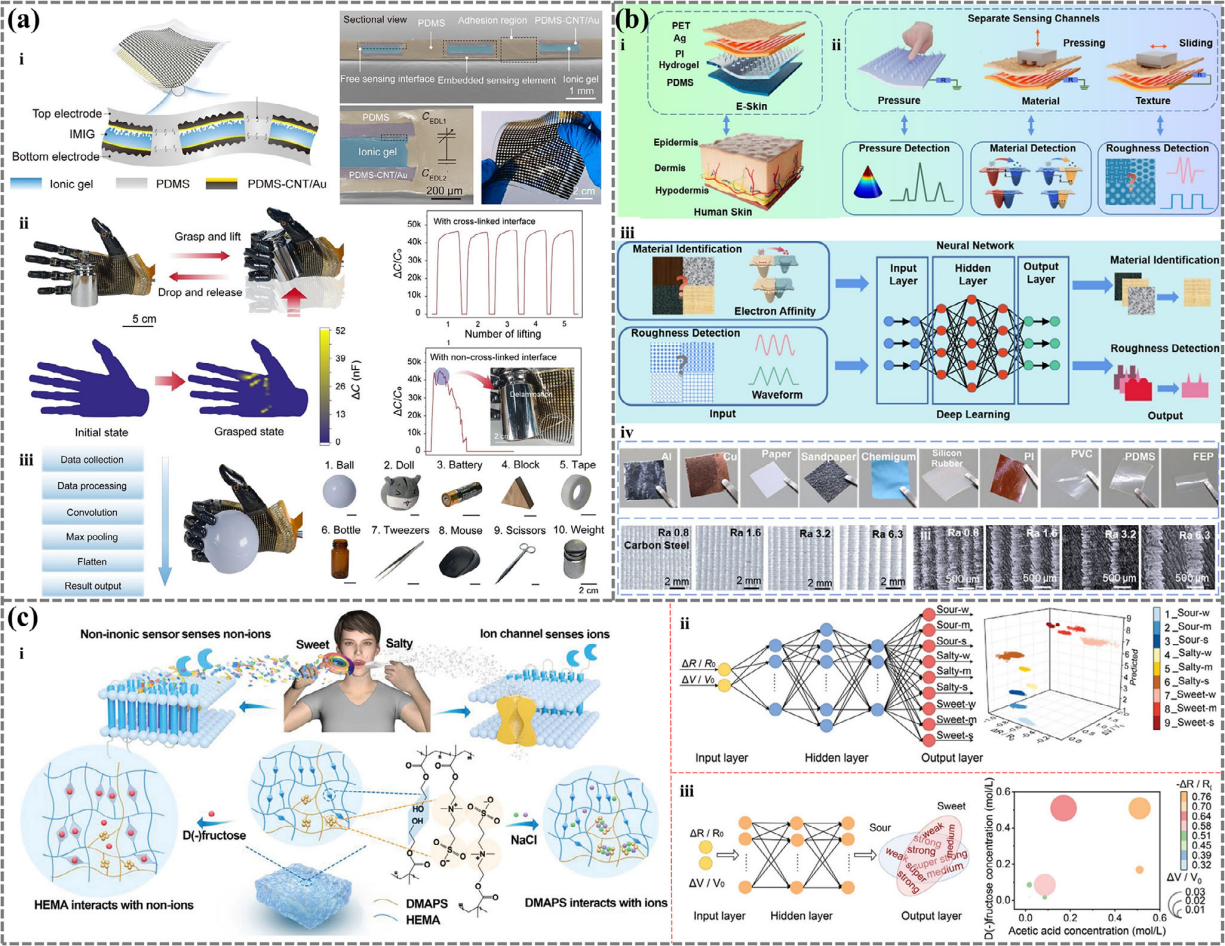
The application of machine learning‐assisted hydrogel and ionogel sensors in object recognition. Iontronic skin with embedded configuration and EDL‐based capacitance‐to‐pressure sensitivity. a i) was used in a prosthetic hand to obtain the pressure mapping data a ii), recognizing 10 objects with machine learning a iii). Reproduced with permission.^[^ ^166^ ^]^ Copyright 2023, The American Association for the Advancement of Science. b) The material and roughness recognition process based on the TENG sensor and machine learning. Reproduced with permission.^[^ ^169^ ^]^ Copyright 2023, American Chemical society. Inspired by the skin, BHES was prepared by assembling wrinkled PET, Ag, PI, PAM/CA DN hydrogel, and PDMS b i). It can sense the material and roughness based on contact electrification and the stick‐slip effect b ii). By using a deep learning neural network model b iii), precise recognition of material types and surface textures b iv) was achieved. c) Taste sensing based on dual‐responsive hydrogel and machine learning. Reproduced with permission.^[^ ^172^ ^]^ Copyright 2023, Wiley. Schematic illustration of both electrical and volumetric responsive sensors made of poly (DMAPS‐co‐HEMA), involving HEMA interacts with nonionic D(‐)‐fructose, while DMAPS associates with ionic NaCl and acids c i). Through feature extraction and machine learning, it enables the recognition of a single taste of sweetness, saltiness, and sourness at weak, medium, and strong levels c ii) and mixed sweetness and sourness c iii).

Besides the pressure‐induced resistance signals, electric double layer (EDL) interfaces can create the pressure‐sensitive capacitive signal, which is promising in improving sensing performance. Shi et al. proposed embedding isolated microstructured ionic gel (IMIG) within a cavity array of PDMS substrate, with lateral cross‐linking of the IMIGs to achieve both high sensitivity and mechanical robustness (**Figure** **8**a i).^[^ ^166^ ^]^ This embedded iontronic sensing element configuration created a distinct iontronic interface, enhancing capacitance‐to‐pressure sensitivity while suppressing signal cross‐talk. When the sensor array was attached to the palm of an artificial hand, real‐time pressure mapping during object grasping was achieved (Figure 8a ii). Utilizing a 1D‐CNN deep learning algorithm, the system successfully recognized 10 different objects with an accuracy of 99.5% (Figure 8a iii).

#### 5.3.2 Triboelectric Nanogenerator (TENG) Based Sensor

Triboelectric sensors demonstrate significant application potential in the field of object recognition, particularly in tactile detection and surface feature identification. These sensors can effectively capture triboelectric signals generated when materials come into contact with the sensor surface, enabling high‐precision recognition of complex objects. Zhang et al. developed a triboelectric nanogenerator (PTSM‐TENG) based on a dual‐network hydrogel (PTSM) constructed from polyacrylamide (PAM), tannic acid (TA), sodium alginate (SA), and MXene by a one‐pot method.^[^ ^52^ ^]^ This hydrogel exhibits excellent stretchability, adhesion, and self‐healing properties. The PTSM‐TENG was attached to the glove and collected triboelectric signals generated by touching five different spherical objects. With the assistance of machine learning, the system can classify and recognize five spherical objects with an accuracy of 98.7%.

Besides hydrogels, ionogels can also be adopted to recognize objects due to their ionic conductivities and superior mechanical properties. PAIG‐TENG, based on polyacrylic acid ionogel (PAIG), was developed, synthesized with liquid metal and graphene oxide nanosheets as initiators and cross‐linkers in monomer suspension.^[^ ^42^ ^]^ The obtained PAIG showed a favorable combination of conductive and mechanical properties, along with self‐healing, antifreezing, and antidrying properties. The PAIG‐TENG sensor was introduced to the intelligent robot system equipped with machine learning for material‐specific ball recognition and sorting. Similarly, Zhong et al. developed an ionogel‐based triboelectric nanogenerator (I‐TENG) sensor composed of self‐developed ionogels, aramid fibers, and silicone tubes.^[^ ^43^ ^]^ This sensor can perceive object information when grasping objects with a robotic hand, where sensors are placed at both the front and back of all five fingers. Using the support vector machine (SVM) method, various objects with different shapes and weights can be classified, achieving 90.38% accuracy for shape and 85.00% for weight.

#### 5.3.3 Multimodal Sensing

Multimodal sensing means simultaneously integrating and processing various types of sensory data, including but not limited to pressure, temperature, and volumetric changes. When integrated with machine learning algorithms, dual‐responsive/multi‐responsive ionogels and hydrogels are particularly well‐suited for multimodal sensing applications, such as object recognition and taste sensing. Lv et al. prepared poly (vinylidene fluoride‐co‐chlorotrifluoroethylene) (PVDF‐co‐CTFE)‐based ionogels (PIG) with microphase‐separated bicontinuous structure and skin‐like mechanics. This PIG was sensitive to both pressure and temperature. An LSTM model was utilized to decouple the voltage signal of the PIG sensor into pressure and temperature, enabling synchronous sensing and monitoring of both pressure and temperature.^[^ ^167^ ^]^ A soft actuator was integrated with two stretchable hydrogel sensors capable of simultaneously detecting mechanical deformation and temperature variations.^[^ ^168^ ^]^ A 1D‐CNN and feedforward neural network model were employed to classify five specific categories: free bending, touch without temperature change, touch by hot objects, twisting, and stretching, highlighting the potential of multimodal sensing in intelligent perception of both deformation modes and thermal stimuli. Another biomimetic, hydrogel‐based electronic skin (BHES) has been reported, composed of a layer of nanoscale wrinkled poly (ethylene terephthalate) (PET), interdigital silver electrodes, and patterned microcone double‐network (DN) hydrogel, designed to mimic the epidermis, internal mechanoreceptor, and dermis of human skin (Figure 8b i).^[^ ^169^ ^]^ The microcone DN hydrogel prepared through a PDMS mold provided high‐performance pressure sensing. When pressed by material, the BHES can recognize the material species based on the voltage signals generated from the electron affinity differences between various materials. During the sliding motion of an object across the BHES, the texture/roughness of the object can be identified via the stick‐slip mechanism by analyzing the generated waveforms (Figure 8b ii,iii). Assisted by a CNN, the BHES achieved 95.00% accuracy in material recognition and 97.20% accuracy in detecting surface roughness (Figure 8b iv). In addition to the hydrogel/ionogel sensors related to hand tasks, electronic skin materials developed from silver nanoparticle ink, when combined with deep learning, enable a single skin sensor to decode complex movements of all five fingers in real time.^[^ ^170^ ^]^ Meanwhile, the integration of Ag@Au core‐shell nanomesh with meta‐learning capabilities allows for rapid adaptation to different users and daily tasks, including motion command recognition, two‐handed keyboard input, and object identification.^[^ ^171^ ^]^

Taste‐sensing abilities are essential for the development of an artificial tongue. Miao et al. developed a bioinspired dual‐responsive hydrogel sensor, synthesized by free‐radical copolymerization of 2‐hydroxyethyl methacrylate (HEMA) and N‐(3‐sulfopropyl)‐N‐(methacryloxyethyl)‐N, N‐dimethylammonium betaine (DMAPS) monomers (Figure 8c).^[^ ^172^ ^]^ Ionic model molecules (e.g., sodium chloride and acetic acid) bond electrostatically with DMAPS domains, while nonionic molecules (e.g., D(‐)‐fructose) form hydrogen bonds with HEMA, triggering complex changes in both resistance and volume (Figure 8c i). With the assistance of a deep neural network (multi‐layer perceptron), the sensor can achieve semi‐quantitative recognition of a single taste between sweetness, saltiness, and sourness with defined concentrations (Figure 8c ii) and mixed sweetness and sourness (Figure 8c iii). This research advanced the design of artificial taste sensors.

### 5.4 Motion Recognition

**Figure 9:**
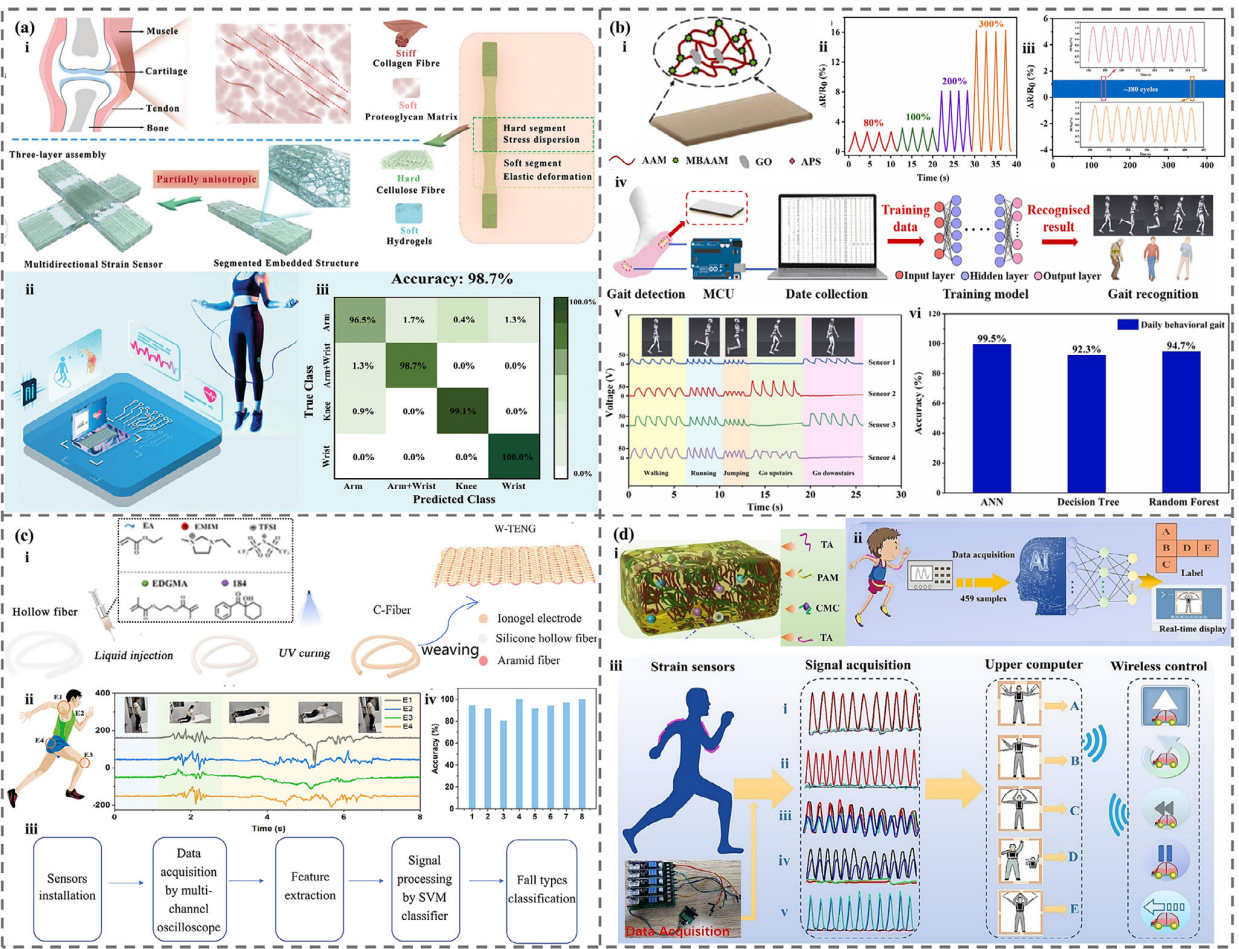
The application of machine learning assisted hydrogel or ionogel sensors in motion recognition. a) Schematic diagram of a wearable multidirectional sensing system constructed by a segmented embedded hydrogel for monitoring joint movement. Reproduced with permission.^[^ ^174^ ^]^ Copyright 2023, Wiley. a i) The polyacrylamide network is used as the matrix part, and the wood is used as the template of the hard part to manufacture the segmented embedded structure. Machine learning and computer programs were used for data processing and joint motion classification, a ii), demonstrating high accuracy a iii). b) Schematic diagram of an intelligent sensor based on graphene oxide and polyacrylamide hydrogel for gait recognition in patients with Parkinsonian's disease and hemiplegia. Reproduced with permission.^[^ ^130^ ^]^ Copyright 2022, Elsevier. b i) Structure diagram of graphene oxide‐polyacrylamide (GO‐PAM) hydrogel. The GO‐PAM showed the responsiveness of relative resistance change within a large tensile strain range b ii) and the stability test of the stretchability b iii). b iv) The gait recognition system based on the GO‐PAM hydrogel and machine learning to monitor five different types of daily human activities b v) as well as to classify Parkinsonian and hemiplegic patients, with the ANN, decision tree, and random forest models to assess the recognition accuracy. c) Ionogel is woven into a flexible sensor for human fall recognition. c i) Synthesis process of composite fiber (C‐fiber) and triboelectric nanogenerator (TENG) sensors based on ionogel. Reproduced with permission.^[^ ^178^ ^]^ Copyright 2023, Elsevier. c ii) Flow chart for collecting and processing sensor data based on SVM. c iii) Train the SVM confusion matrix using the original data feature data. c iv) Summary chart of the eight confusion matrix accuracy. d) Schematic diagram of a dual‐network polyacrylamide hydrogel strain sensor used to control wireless vehicles. Reproduced with permission.^[^ ^22^ ^]^ Copyright 2023, Elsevier. d i) Schematic diagram of the cross‐linking network structure of polyacrylamide hydrogel. d ii) Schematic diagram of classification and recognition of motion signals detected by the hydrogel sensor. d iii) Flowchart for motion recognition and remote control of intelligent wearable systems, which uses machine learning to classify motion signals into five categories: A, B, C, D, and E, for controlling the straight, turning, backward, stopping, and forward movements of wireless vehicles.

Machine learning is good at rapidly classifying signals generated by sensors that detect human movements. As a result, the combination of machine learning with hydrogel and ionogel sensors has found widespread applications in motion recognition.^[^ ^173^ ^]^ Real‐time motion analysis is employed for monitoring daily activities and sports. Inspired by the staggered structure of cartilage, Jiang et al. integrated soft and hard materials (i.e., PAAm matrix and chemically treated wood) to construct segmented embedded hydrogel sensors through topological design and zipper shear chain technology (**Figure** **9**a i).^[^ ^174^ ^]^ This sensor can accurately capture the multi‐directional strain and pressure changes in the plane, and realize the real‐time continuous monitoring of high strain, multi‐degree of freedom joint motion (Figure 9a ii). With the support vector machine learning algorithm, the sensor successfully classified and recognized diverse motion poses and joint activity states with a high accuracy of 98.7% (Figure 9a iii). Unlike hydrogel, the ionogel shows thermal stability behavior under a wide temperature window, showing the potential to recognize human motion in cold environments.^[^ ^175^ ^]^ Yang et al designed a stretchable double network organic ionogel based on ethylene glycol, exhibiting antifreezing properties.^[^ ^176^ ^]^ These ionogel sensors were deployed on the athlete's left elbow, right elbow, right forearm, waist, knees, and right ankle to collect the resistance changes under different strains. The CNN model can analyze the sensor signal in real time, accurately assess the athletes' sports state, and realize real‐time state tracking during winter sports. Moreover, a serpentine‐shaped ionogel was made under a patterned mask with UV‐induced polymerization of mixed poly (ethylene glycol) diacrylate (PEGDA), acrylic acid (AA), and varying IL ratios of 1‐ethyl‐3‐methylimidazolium bis(trifluoromethylsulfonyl)‐imide (EMIM‐TFSI).^[^ ^177^ ^]^ When attached to different parts of joints, such as the wrist, fingers, elbows, and shoulders, it can capture the resistance or capacitance changes caused by human movements. By using an artificial neural network (ANN) algorithm for data training across different strains and temperature stimuli, the system successfully measured and predicted joint movements under varying environmental temperature conditions, achieving adaptive human gesture recognition.

Gait recognition is essential for physical training and health monitoring. Wang et al. prepared an intelligent sensor based on graphene oxide and polyacrylamide hydrogel (Figure 9b i).^[^ ^130^ ^]^ The relative resistance of the graphene oxide‐polyacrylamide hydrogel exhibits stable variations within a large tensile strain range (Figure 9b ii), and it possesses excellent durability, capable of generating consistent changes in relative resistance under 380 cycles (Figure 9b iii). Researchers have designed an intelligent insole system composed of a hydrogel, a PC interface, and a data processing module (Figure 9b iv). ANN, decision tree, and random forest algorithms were utilized to efficiently recognize daily gait activities (walking, running, jumping, going upstairs, and downstairs) (Figure 9b v), as well as pathological gait (Parkinson's disease, left hemiplegia, and right hemiplegia). The ANN algorithm achieved accuracy rates of 99.5% for daily gait recognition and 98.2% for pathological gait recognition. (Figure 9b vi). This system greatly facilitates gait recognition, as well as early diagnosis and evaluation of patients.

Motion analysis and monitoring also play a critical role in safety assurance, such as detecting accidental falls in daily activities^[^ ^178^, ^179^ ^]^ and monitoring the driver's state.^[^ ^180^ ^]^ For the human accidental falling detection, the ionogel precursor was injected into the hollow fiber tube and prepared conductive fiber after UV curing (Figure 9c i).^[^ ^178^ ^]^ These fibers were woven into a flexible W‐TENG sensor designed to monitor human behavior with high accuracy, utilizing a support vector machine (SVM) to train original motion data (Figure 9c ii,iii). The column graph of the confusion matrix with eight features exhibited that the model trained using the standard deviation as a statistical feature was the best for sample prediction (Figure 9c iv). Ultimately, this model achieved 100% accuracy in identifying different fall categories, enabling real‐time monitoring of fall situations during walking. Hydrogel's excellent biocompatibility enables the same applications in infant care. Guo et al. designed a body area triboelectric sensor network based on agar hydrogel for infant motion monitoring.^[^ ^179^ ^]^ This approach ensures that all materials used in the soft sensors are edible, effectively mitigating the risks and consequences of accidental ingestion by infants. Eleven triboelectric sensors are attached to the baby, and a deep learning algorithm is used to analyze the generated motion signals for the monitoring of infant motion states, including falling forward, falling backward, turning over, being held, patting, and applauding. Multi‐dimensional information detection of drivers is crucial to ensure safety. Luo et al. increased the sensor performance by doping NaCl solution in PVA hydrogel and introducing a curved interface between the friction layer and the electrode.^[^ ^180^ ^]^ An intelligent neck ring based on the sensor is designed to collect signals of neck muscle movement, including turning the head, nodding, coughing, and speaking. The KNN, SVM, and CNN algorithms are employed to evaluate and classify the driver's concentration, forming a comprehensive monitoring of the driver's status.

Sensors based on hydrogels and ionogels have been reported to be used in remote control of unmanned aerial vehicles,^[^ ^169^ ^]^ intelligent cars,^[^ ^21^, ^22^ ^]^ robot hands^[^ ^160^, ^162^, ^164^, ^181^, ^182^ ^]^ and wheelchairs.^[^ ^183^ ^]^ A convenient unmanned aerial vehicle (UAV) control technology was realized by integrating the signal acquisition and wireless signal transmission circuit with the proposed double network hydrogel.^[^ ^169^ ^]^ It enabled the conversion of subtle finger movements into electrical signals and real‐time control of UAV swarms. Leveraging the characteristics of ionogel, control of robotic hands can be achieved even under extreme conditions. Hao et al. prepared a self‐healable ionogel via polymerization of zwitterionic ionic liquid (3‐dimethyl (methacryloyloxyethyl) ammonium propane sulfonate, DMAPS) and acrylic acid (AA) in 1‐ethyl‐3‐methylimidazolium ethyl sulfate (EMIMEtSO_4_), with chemical macro‐cross‐linkers (acrylate‐terminated hyperbranched polyester polyols).^[^ ^181^ ^]^ This ionogel can adhere to the skin and the surface of gloves, which can be used for real‐time wireless control of robot hands under extreme conditions, such as high vacuum, high and low temperatures. Besides, a 3D printable ionogel was prepared through the polymerization of polyhedral oligomeric silsesquioxanes (POSS), AA, and EMIM(EtO)_2_P_2_, where POSS acts as a chemical crosslinker and PAA hydrogen bonds as reversible physical crosslinkers.^[^ ^182^ ^]^ With these specialized dual crosslinking structures, the ionogel‐based human‐machine interface operated effectively at extreme temperatures ranging from −60 to 150 °C, promptly responding to hand movements and controlling the mechanical hand.

Machine learning has been added to enhance the recognition ability of sensors for human actions, and then the classification of commands from human intention to realize more precise machine control and human‐machine interaction. Li et al. designed a PAM/CMC/TA (PCT) hydrogel with tannic acid (TA) as a physical cross‐linker to reinforce polyacrylamide (PAM) and sodium carboxymethyl cellulose (CMC) networks (Figure 9d i). Classification and recognition of motion signals detected by the hydrogel sensor enabled the control of a small vehicle (Figure 9d ii). The movement signal is collected by wearing the sensor on the shoulder and elbow. The LSTM model in machine learning processes the signal and outputs it as different command signals, which further control the wireless vehicle to forward, backward, turn, and stop actions (Figure 9d iii).^[^ ^22^ ^]^ In addition, one hydrogel biosensor was fixed on the forehead of wheelchair users.^[^ ^183^ ^]^ The collected signal was used to analyze the eye movement through a wavelet transform support vector algorithm, working as a human‐machine interface to realize the accurate control of the wheelchair. This eye movement control method is significant for the paralyzed population.

### 5.5 Health Monitoring

**Figure 10:**
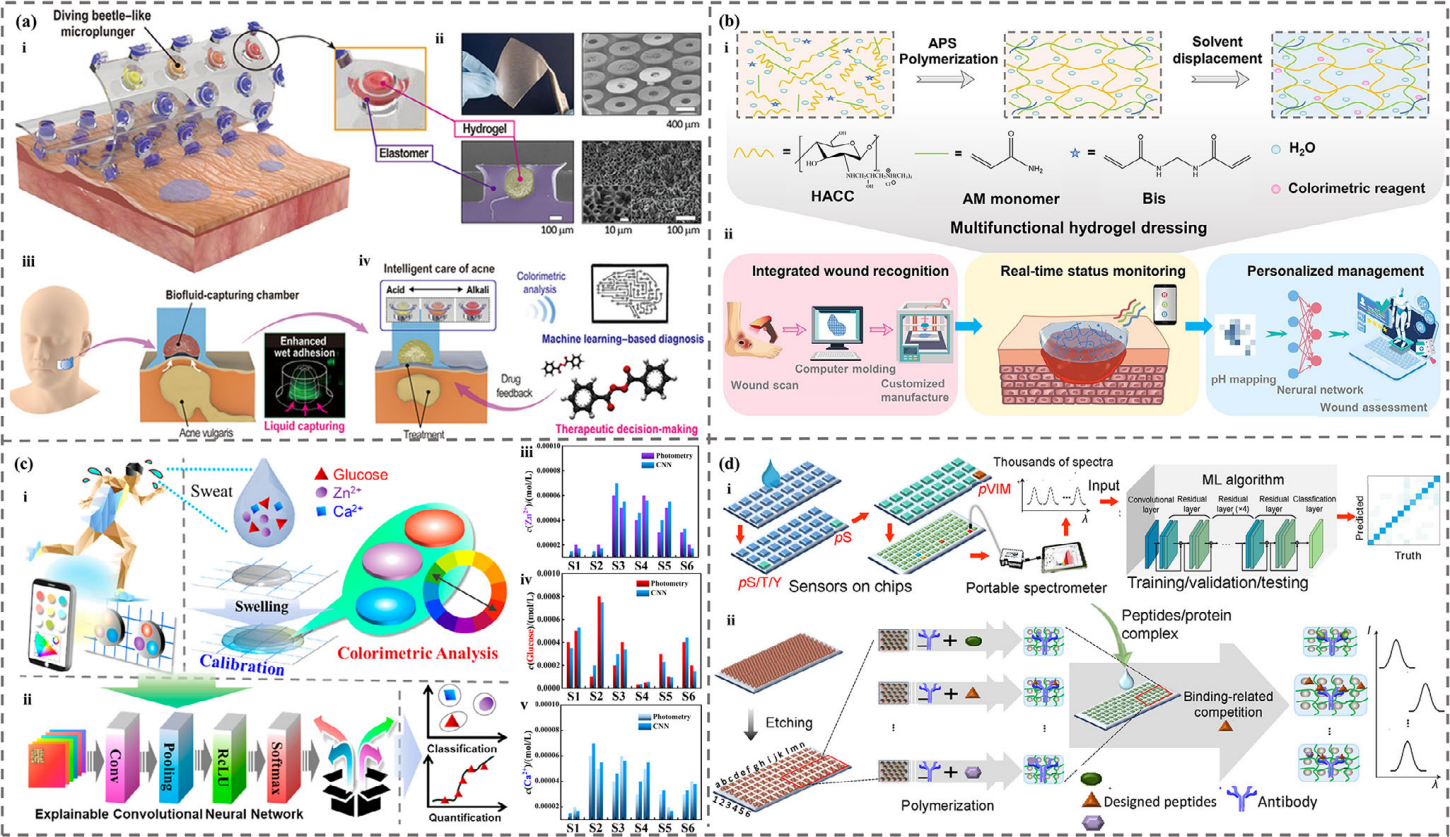
The application of machine learning assisted hydrogel or ionogel sensors in monitoring physiological signals or biomarkers. a) Schematic diagram of integrated equipment composed of microplungers and polyacrylamide hydrogels used for skin disease management. Reproduced with permission.^[^ ^185^ ^]^ Copyright 2021, The American Association for the Advancement of Science. b) Schematic diagram of multifunctional hydrogel prepared by polyacrylamide and chitosan quaternary ammonium salt as a wound dressing for intelligent wound monitoring. Reproduced with permission.^[^ ^53^ ^]^ Copyright 2022, Elsevier. c) Schematic showing the colorimetric detection of sweat biomarkers, such as glucose, assisted by the CNN model. Reproduced with permission.^[^ ^190^ ^]^ Copyright 2024, American Chemical Society. d) Schematic diagram of a deep learning assisted hydrogel sensor for point‐of‐care identification of post‐translational modifications. Reproduced with permission.^[^ ^193^ ^]^ Copyright 2023, Wiley.

Real‐time monitoring of physiological information is important for health management, disease diagnosis, and treatment. The integration of machine learning with gel‐based flexible sensors improves both individual comfort and testing accuracy compared to traditional devices.^[^ ^44^, ^184^ ^]^ Hydrogel sensors can act directly on the skin due to their good biocompatibility. By enhancing the data analysis and processing of sensors through machine learning, effective skin disease and wound management can be achieved.^[^ ^53^, ^185^, ^186^ ^]^ Inspired by the diving beetle's microplunger, researchers designed a suction‐mediated device composed of microplungers and polyacrylamide (PAAm) hydrogels to improve the adhesion and biofluid capture capabilities (**Figure** **10**a i),^[^ ^185^ ^]^ exhibiting the structural uniformity of the suction chamber array (Figure 10a ii). The pH‐responsive phenol red was embedded into the PAAm hydrogel, enabling easy biofluid capture and intelligent pH monitoring in acne‐affected skin areas using machine learning techniques (Figure 10a iii). This approach improves the efficiency and accuracy of the therapeutic feedback for acne treatment. Wang et al. loaded the colorimetric reagent (litmus) into the hydrogel composed of PAAm and chitosan quaternary ammonium salt to create a multifunctional hydrogel dressing with excellent antibacterial, hemostatic, and adhesive properties, along with pH colorimetric detection capabilities (Figure 10b i).^[^ ^53^ ^]^ A personalized wound dressing can be customized by scanning the wound and using 3D printing to precisely replicate the wound contour. Subsequently, a convolutional neural network (CNN) was utilized to analyze the RGB values of the hydrogel patch to predict its pH value, which reflects the status of the wound. This method achieved a prediction accuracy of 94.47%, enabling intelligent wound management (Figure 10b ii). A binary wearable system, consisting of an AI‐guiding wearable sensor and a smart wound dressing bandage, is designed for use by clinical professionals and patients, respectively.^[^ ^186^ ^]^ This intelligent bandage, based on poly (vinyl acrylic) gel@PANI/Cu_2_O NPs wound dressing, generated pH‐responsive currents during wound healing. The ANN algorithm‐assisted wearable sensor can achieve 94.6% accuracy in classifying the inflammation, proliferation, and remodeling stages of wound healing in patients with skin diseases, thereby enabling effective contactless chronic skin monitoring.

In addition to skin diseases and wound management, machine learning combined with gel sensors has been effectively applied in cardiovascular disease monitoring. An atrial fibrillation prediction wristband (AFPW) consisted of polyvinylidene fluoride (PVDF) piezoelectric film as the sensing layer and the hydrogel as the bonding layer, enhancing skin affinity and improving user experience.^[^ ^187^ ^]^ By applying linear discriminant analysis (LDA) to analyze the collected pulse wave signals, the wristband can predict atrial fibrillation with an accuracy of 91%.

Furthermore, hydrogel sensors can be used to monitor various biomarkers or physiological signals within the human body, such as pH levels, glucose, chloride ions, and calcium ions in sweat.^[^ ^188^, ^189^ ^]^ The integration with machine learning technology significantly improves the accuracy of predictions. The embedded polyacrylate sodium‐poly(vinyl alcohol) composite hydrogel, embedded with enzyme/indicator, with colorimetric sensing and sweat absorbing abilities (Figure 10c), was designed to classify and quantify the detect the, glucose in sweat, assisted by the CNN model.^[^ ^190^ ^]^ Sweat electrochemical biosensors, iontophoresis electrodes, microfluidics technology, and energy storage units were integrated for simultaneous monitoring of glucose, alcohol, pH, temperature, and heart rate.^[^ ^191^ ^]^ Researchers also predicted the degree of behavioral impairments in individuals after alcohol consumption with the introduction of machine learning.

Numerous human diseases, including but not limited to cancers, are related to aberrant post‐translational modifications (PTMs). Therefore, the point‐of‐care (POC) identification of PTMs offers significant potential for disease diagnosis.^[^ ^192^ ^]^ Recently, the multiplexed photonic crystal hydrogel (PCH) sensor array was immobilized with different antigen‐antibody pairs (Figure 10d i),^[^ ^193^ ^]^ enabling the generation of spectral data. Analyzing and processing the spectral data and color information obtained from sensors using CNNs makes it possible to visually detect PTMs in recombinant proteins and complex biological samples (Figure 10d ii). This enables the identification of PTMs and the accurate prediction of PTM concentrations, which is significant for protein‐related disease diagnosis. In contrast to the non‐invasive sensing described above, PEDOT:PSS hydrogel‐based implantable devices^[^ ^194^, ^195^, ^196^, ^197^ ^]^ enable direct monitoring of electrophysiological signals from the brain and heart. However, this advantage introduces critical challenges in miniaturization, biocompatibility, and long‐term signal stability. Although the integration of implantable sensors with machine learning algorithms remains relatively unexplored, it constitutes a research area of critical importance, especially for the precise diagnosis and active therapy at the implant site.

### 5.6 Other Applications

#### 5.6.1 Throat Speech Recognition and Larynx Diseases Detection

In machine learning assisted sensors, throat vibration information can be extracted and analyzed to interpret the speech or detect abnormalities of individuals, offering a novel communication method for aphasia and a diagnosis of laryngeal cancer and other diseases that affect the larynx movement.

**Figure 11:**
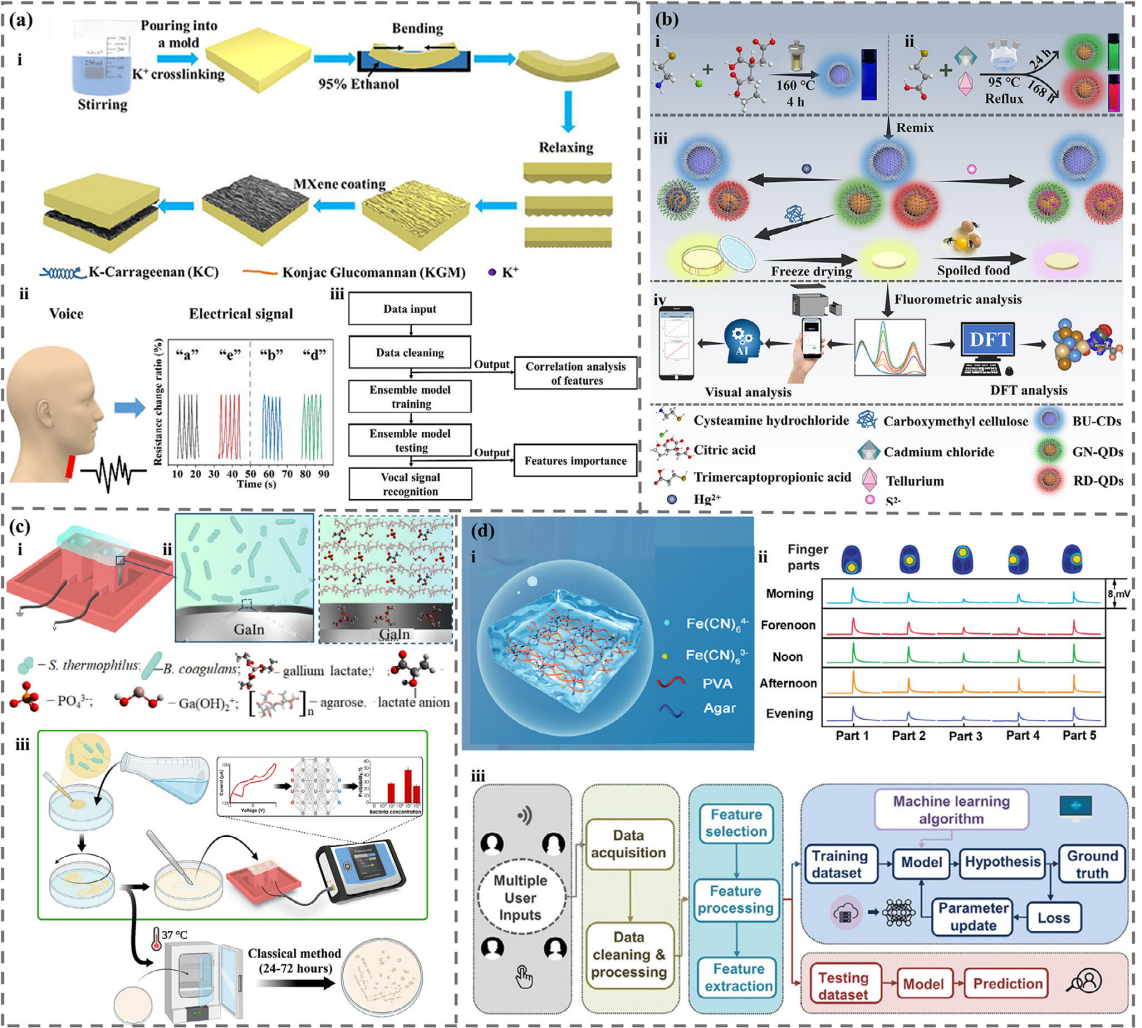
Other applications of machine learning assisted hydrogel/ionogel sensors in speech recognition, food testing, and identity recognition. a) Schematic diagram of a composite hydrogel with wrinkle structure embedded with MXene for throat speech recognition. Reproduced with permission.^[^ ^54^ ^]^Copyright 2023, American Chemical Society. A biomimetic hydrogel with an embedded wrinkled structure is made by surface buckling a i). Resistance signal data are collected a ii) and trained by XGBoost model a iii), successfully demonstrating the ability to distinguish the pronunciation of four letters "a", "b", "e", and "d". b) The schematic diagram of detecting the content of and sulfide in food by combining hydrogel probes that can respectively respond to and sulfide with fluorescence changes. Reproduced with permission.^[^ ^201^ ^]^Copyright 2023, American Chemical Society. The blue‐emission carbon quantum dots (BU‐CDs) b i) and red and green fluorescent CdZnTe quantum dots (namely, RD‐QDs and GN‐QDs, specifically) b ii) are prepared by the hydrothermal method. Combined with smartphone photography, machine learning, and DFT analysis, it can achieve the detection of sulfide content in food b iii, iv). c) Schematic diagram of detection of *S. thermophilus* and *B. coagulans* (two kinds of commonly used bacteria in milk production) based on soft hydrogel /eutectic gallium‐indium alloy (eGaIn) interface. Reproduced with permission.^[^ ^55^ ^]^ Copyright 2022, American Chemical Society. c i) Scheme of the electrochemical system. c ii) Interaction of lactate anions (metabolites of *S. thermophilus* and *B. coagulans*) with eGaIn to form insoluble films, which induced the changes of current‐voltage signals. c iii) Process diagram of machine learning‐assisted electrochemical platform for bacterial concentration detection. d) The inherent temperature features of five distinct positions of fingers identified by the thermogalvanic hydrogel sensor are used for identification recognition. Reproduced with permission.^[^ ^56^ ^]^ Copyright 2024, Wiley. d i)Structure diagram of thermogalvanic hydrogel. d ii) The electrical signals from thermogalvanic hydrogel arrays across different finger parts of users at different times. d iii) Flowchart for identity recognition assisted by machine learning.

To enhance the sensitivity to subtle throat vibrations, Chen et al., inspired by the microstructure of skin, developed a composite hydrogel with an embedded wrinkle structure composed of konjac glucomannan (KGM), k‐carrageenan (KC), and Mxene (**Figure** **11**a i). When applied to the throat, this sensor effectively detected resistance signals generated during the pronunciation of “a”, “e”, “b”, and “d” (Figure 11a ii). By combining the sensor with the XGBoost algorithm, it successfully distinguished between the pronunciations of “a”, “e”, “b”, and “d” (Figure 11a iii).^[^ ^54^ ^]^ Zhou et al. incorporated the graphene oxide modified by polydopamine@Ag (PDA@Ag) nanoparticles into a dual‐network hydrogel.^[^ ^198^ ^]^ The resulting hydrogel‐based strain sensor was used to detect subtle throat voice movements and to further classify the throat signals using a transfer deep learning algorithm based on the Resnet50 neural network. The system successfully recognized 10 commonly used words for patient communication (e.g., “pain”, “pee”, “ache”, “fever”, “help”, “doctor”, and “water”), demonstrating potential for enabling individuals with aphasia to interact with their surroundings.

The vibrations and movements of the throat can be used not only for speech recognition but also for the diagnosis and rehabilitation of diseases that influence the larynx movement.^[^ ^199^ ^]^Xu et al. prepared a composite hydrogel based on DMAPS and ionic salt with a polydimethylsiloxane skeleton.^[^ ^200^ ^]^ A laryngeal patch fabricated from this hydrogel can detect the vibrations and muscle activities of the throat continuously and noninvasively. By employing a CNN to process the data, researchers can effectively evaluate and classify the features of both healthy individuals and patients with laryngeal cancer and myasthenia gravis. This approach has the potential to accelerate the diagnosis, treatment, and rehabilitation of various diseases.

#### 5.6.2 Food Testing

Gel‐based sensors can be utilized in food detection in various ways, particularly in detecting pathogens, hazards, contaminants, and spoilage indicators to ensure their safety and freshness for public health.^[^ ^202^, ^203^ ^]^ Machine learning‐assisted hydrogel sensors based on chromatic indicators provide high‐accuracy monitoring of food safety. For example, a polychromatic probe, comprising blue fluorescent carbon quantum dots (BU‐CDs) (Figure 11b i),^[^ ^201^ ^]^ red and green fluorescent quantum dots (abbreviated as RD‐QDs and GN‐QDs, respectively), was prepared using a hydrothermal method (Figure 11b ii). The multicolor probe was combined with a hydrogel, enabling visual monitoring of food freshness (Figure 11b iii). By integrating the probe‐based hydrogel film with a smartphone device equipped with machine learning techniques, a more accurate linear relationship between the color change and the concentration of the detected substance was achieved, enabling quantitative detection of harmful Hg^2+^ and sulfide both in water and food (Figure 11b iv). Similarly, Cui et al. designed a sodium alginate‐co‐pigment composite hydrogel, with the chromogenic reaction between anthocyanins of blueberry and bilberry and biogenic amines (products of food spoilage) to test the freshness of fish assisted by a genetic algorithm back propagation model (GA–BP).^[^ ^204^ ^]^

Besides chromatic indicators, electrical signals generated during the metabolic process are also useful for the food industry, especially in dairy products. A bio‐electrochemical system consists of two eutectic gallium–indium alloy (eGaIn) electrodes and a hydrogel, which is made from phosphate‐buffered saline (PBS) and agar to ensure the viability of bacteria during detection (Figure 11c i). Within the interface of hydrogel and eGaIn electrodes, eGaIn reacted with the metabolite of lactic acid produced by different concentrations of *S. thermophilus* and *B. coagulans* to form an insoluble film (Figure 11c ii), and thus generated different current‐voltage curves. The collected data are processed and predicted by a multilayer perceptron model with high accuracy. This machine learning‐assisted hydrogel‐based automated sensing platform achieved rapid concentration detection of bacteria, reducing the traditional detection time from 3 days to just 15 min (Figure 11c iii).^[^ ^55^ ^]^

#### 5.6.3 Identity Recognition

Identity recognition based on biometric features plays a crucial role in both physical security and digital safety. Ma et al. designed a thermoelectric hydrogel sensor for identity recognition.^[^ ^56^ ^]^ This thermoelectric hydrogel is prepared by a physical crosslinking method to form a double‐network PVA/agar hydrogel with /glycerol as a binary solvent, absorbing as the redox couple (Figure 11d i). Only a simple touch behavior was required to achieve identity recognition. The voltage signals changed upon a temperature difference from the touched finger. Sufficient features of the voltage changes are extracted, including but not limited to five typical positions of a finger touch at different times (Figure 11d ii). By analyzing these electrical signals using a random forest algorithm to construct an identity recognition model, the system can ultimately identify 5 users with a final average accuracy of 97.6% (Figure 11d iii).

## 6 Conclusion and Perspectives

In recent years, hydrogels and ionogels have been extensively utilized in the field of soft sensors due to their unique mechanical properties, flexibility, and biocompatibility. Machine learning, through automatically processing and analyzing large datasets, has the potential to significantly improve the accuracy and efficiency of material design, property prediction, and performance optimization for hydrogels and ionogels. Moreover, it can advance their multifunctional capabilities and expand the applications of sensors fabricated from these materials.

The combination of machine learning and gels has brought significant advantages to the preparation, property prediction, and optimization of hydrogels.^[^ ^205^ ^]^ By establishing and analyzing material databases, machine learning models can extract key features from large datasets to predict and optimize gelation capabilities, mechanical properties, chemical stability, biocompatibility, photosensitivity, bioadhesion, and other characteristics of hydrogels. This enables rapid screening and identification of materials with desired properties while reducing the number of experiments and material consumption. Besides, this data‐driven design approach not only accelerates the finding and development of novel hydrogels but also enhances the performance of hydrogels significantly.

The integration of machine learning algorithms with hydrogel and ionogel sensors has achieved significant progress in various sensing applications. These sensors can detect subtle physical, biological, physiological, or environmental information by monitoring signals such as resistance, voltage, and mechanical strain. Machine learning models are used to process and analyze the captured complex signals, thereby improving the sensors' response accuracy and sensing capabilities. This combination enables tasks such as gesture/handwriting/object/motion recognition, health monitoring, and food detection, providing intelligent solutions for wearable devices, medical equipment, and HMI.

While the integration of machine learning and hydrogel/ionogel sensors has achieved great success, there are still some challenges and limitations.

1. While the machine learning‐assisted design of hydrogels has been explored, there are almost no reports on the integration of machine learning with the preparation of ionogels.^[^ ^206^ ^]^ More attention is needed to accelerate the design, performance optimization, and prediction for both hydrogels and ionogels.
2. In the field of gesture recognition, the scope of gesture recognition is limited to a range of several distinct gestures. Continuous, precise, and synchronous recognition needs to be developed for more complex movements and gestures. Additionally, the potential introduction of computer vision,^[^ ^207^ ^]^ natural language processing, and artificial intelligence‐generated content should be explored to directly translate these gestures into multiple forms of output, such as text, sound, images, and video. This approach could offer more comprehensive solutions for facilitating communication between deaf, mute, blind communities and non‐disabled individuals.
3. In the healthcare field, integrated all‐in‐one sensing systems that capture physical, biological, and physiological signals should be developed and combined with the Internet of Things (IoT) and cloud computing. This integration is essential for enabling real‐time, long‐term, all‐around health management, as well as facilitating intelligent diagnosis, treatment, and prognosis management. For example, with the help of these advanced systems, medical teams can develop personalized surgical plans, conduct virtual surgery simulations, and precisely monitor and manage intraoperative and postoperative conditions to optimize treatment outcomes.
4. Additionally, there are a few reports on machine learning‐assisted sensors with neuroscience. Hydrogel and ionogel sensors have the potential to work as electrophysiological interfaces for neuroscience research and the treatment of neurological disorders. Thus, developing highly adaptive and biocompatible neural interfaces and brain‐computer interfaces based on these sensors will become a key area of future research.
5. There are several challenges on the machine learning side, such as experimental data processing, model generalization, model optimization, and data privacy and security. First, gel sensors interact with the human body in highly complex and dynamic conditions, usually resulting in noisy signals. To address this, more efficient data preprocessing and feature extraction algorithms are needed to enhance signal clarity and usability. Second, the processes of data acquisition typically require extensive experimentation, which is both costly and time‐consuming. Consequently, the size of available training datasets is often limited, potentially impairing the model's generalization capabilities and leading to inconsistencies between experimental results and real‐world performance. Third, the computational efficiency of machine learning models is crucial. These models need to be optimized to function effectively within the resource constraints of practical applications. Finally, privacy‐preserving algorithms and secure data storage solutions are essential to protect personal information in everyday use.

Overall, machine learning and gel‐based sensor materials have a complementary and mutually beneficial relationship. Machine learning optimizes the design and performance of gels and accelerates the development of novel gels. At the same time, the unique properties of hydrogels and ionogels provide a robust data foundation for machine learning algorithms, further improving their accuracy and extending their capabilities. The synergy between machine learning and sensors based on hydrogels/ionogels has accelerated the development of smart sensors, which are expected to be widely applied in health management, environmental monitoring, pattern recognition, user identification, and other fields. This integration will enable more precise monitoring, intelligent decision support, and personalized services, driving comprehensive innovation and breakthroughs across related industries, such as healthcare, smart wearable devices, and human‐machine interaction.

## Conflict of Interest

The authors declare no conflict of interest.

## References

[1] C. Son , J. Kim , D. Kang , S. Park , C. Ryu , D. Baek , G. Jeong , S. Jeong , S. Ahn , C. Lim , Nat. Commun. 2024, 15, 8003. 39266523 10.1038/s41467-024-52331-4 PMC11393463

[2] R. Yin , D. Wang , S. Zhao , Z. Lou , G. Shen , Adv. Funct. Mater. 2021, 31, 2008936.

[3] J. Wang , W. Lin , Z. Chen , V. O. Nikolaeva , L. O. Alimi , N. M. Khashab , Nat. Commun. 2024, 15, 1575. 38383478 10.1038/s41467-024-46071-8 PMC10881501

[4] J. Xue , Y. Zou , Y. Deng , Z. Li , EcoMat 2022, 4, 12209.

[5] X. Wang , H. Ji , L. Gao , R. Hao , Y. Shi , J. Yang , Y. Hao , J. Chen , Chem. Eng. J. 2024, 495, 153382.

[6] J. T. Glazar , V. B. Shenoy , Nat. Mach. Intel. 2022, 4, 194.

[7] Y. Ling , T. An , L. W. Yap , B. Zhu , S. Gong , W. Cheng , Adv. Mater. 2020, 32, 1904664. 10.1002/adma.201904664 31721340

[8] K. R. Pyun , K. Kwon , M. J. Yoo , K. K. Kim , D. Gong , W.‐H. Yeo , S. Han , S. H. Ko , Natl. Sci. Rev. 2024, 11, nwad298. 38213520 10.1093/nsr/nwad298 PMC10776364

[9] S. Chen , Y. Zhang , Y. Li , P. Wang , D. Hu , Nano Energy 2024, 124, 109443.

[10] J. Oh , S. Kim , S. Lee , S. Jeong , S. H. Ko , J. Bae , Adv. Funct. Mater. 2021, 31, 2007772.

[11] J. J. Park , S. Hong , Y. Jung , P. Won , C. Majidi , M. Kim , S. H. Ko , Adv. Funct. Mater. 2025, 35, 2505089.

[12] M. Kim , J. J. Park , C. Cho , S. H. Ko , Adv. Funct. Mater. 2023, 33, 2303286.

[13] M. Qiang , X. Wang , B. Cheng , W. He , X. Huang , G. Huang , L. Wang , S. K. Ravi , X. Yao , Adv. Funct. Mater. 2025, 08954.

[14] I. Hong , S. Lee , D. Kim , H. Cho , Y. Roh , H. An , S. Hong , S. H. Ko , S. Han , Nanotechnology 2019, 30, 074001. 30523977 10.1088/1361-6528/aaf35c

[15] J. Jin , X. Geng , Q. Chen , T.‐L. Ren , Nano‐Micro Lett. 2022, 14, 64. 10.1007/s40820-022-00793-w PMC8866629 35199258

[16] H. Yoon , J. Choi , J. Kim , J. Kim , J. Min , D. Kim , S. Jeong , J. G. Lee , J. Bang , S. H. Choi , Y. Jeong , C.‐Y. Kim , S. H. Ko , Adv. Funct. Mater. 2024, 34, 2313504.

[17] J. Choi , J. Min , D. Kim , J. Kim , J. Kim , H. Yoon , J. Lee , Y. Jeong , C. Y. Kim , S. H. Ko , ACS Nano 2023, 17, 17966. 37668160 10.1021/acsnano.3c04292

[18] K. K. Kim , J. Choi , J.‐H. Kim , S. Nam , S. H. Ko , Adv. Funct. Mater. 2022, 32, 2106329.

[19] F. Li , Y. Bu , G.‐F. Han , H.‐J. Noh , S.‐J. Kim , I. Ahmad , Y. Lu , P. Zhang , H. Y. Jeong , Z. Fu , Q. Zhong , J.‐B. Baek , Nat. Commun. 2019, 10, 2623. 31197162 10.1038/s41467-019-10622-1 PMC6565687

[20] H. Park , S. Yoon , J. Bang , J. Ahn , G. Choi , D. Kim , J. Min , J. Shin , S. H. Ko , ACS Nano 2025, 19, 30961. 40790995 10.1021/acsnano.5c07646 PMC12410051

[21] W. Wang , H. Zhou , Z. Xu , Z. Li , L. Zhang , P. Wan , Adv. Mater. 2024, 36, 2401035. 10.1002/adma.202401035 38552161

[22] W. Li , S.‐M. Li , M.‐C. Kang , X. Xiong , P. Wang , L.‐Q. Tao , Int. J. Biol. Macromol. 2024, 254, 127434. 37838111 10.1016/j.ijbiomac.2023.127434

[23] S. Mandal , A. Seth , V. Yadav , S. Kumari , M. Kumar , U. Ojha , ACS Appl. Polym. Mater 2019, 2, 618.

[24] J.‐C. Hsieh , H. Alawieh , Y. Li , F. Iwane , L. Zhao , R. Anderson , S. I. Abdullah , K. W. K. Tang , W. Wang , I. Pyatnitskiy , Biosens. Bioelectron. 2022, 218, 114756. 36209529 10.1016/j.bios.2022.114756

[25] H. Guo , M. Bai , Y. Zhu , X. Liu , S. Tian , Y. Long , Y. Ma , C. Wen , Q. Li , J. Yang , Adv. Funct. Mater. 2021, 31, 2106406.

[26] A. López‐Díaz , A. S. Vázquez , E. Vázquez , ACS Nano 2024, 18, 20817. 39099317 10.1021/acsnano.3c12200 PMC11328171

[27] S. Duan , Q. Shi , J. Hong , D. Zhu , Y. Lin , Y. Li , W. Lei , C. Lee , J. Wu , ACS Nano 2023, 17, 1355. 10.1021/acsnano.2c09851 36629247

[28] Z. Wang , L. Xu , W. Liu , Y. Chen , Q. Yang , Z. Tang , H. Tan , N. Li , J. Du , M. Yu , Int. J. Biol. Macromol. 2024, 276, 133802. 38992552 10.1016/j.ijbiomac.2024.133802

[29] Y. Liu , L. Wang , Y. Mi , S. Zhao , S. Qi , M. Sun , B. Peng , Q. Xu , Y. Niu , Y. Zhou , J. Mater. Chem. C 2022, 10, 13351.

[30] C. Wang , Y. Liu , X. Qu , B. Shi , Q. Zheng , X. Lin , S. Chao , C. Wang , J. Zhou , Y. Sun , Adv. Mater. 2022, 34, 2105416. 10.1002/adma.202105416 35103354

[31] L. Wang , T. Xu , X. Zhang , TrAC, Trends Anal. Chem. 2021, 134, 116130.

[32] Y. Wang , X. Jiang , X. Li , K. Ding , X. Liu , B. Huang , J. Ding , K. Qu , W. Sun , Z. Xue , Mater. Horiz. 2023, 10, 4033. 37522298 10.1039/d3mh00326d

[33] X. Li , F. Sun , ACS Appl. Mater. Interfaces 2023, 15, 37717. 37523492 10.1021/acsami.3c06894

[34] J. Kim , J. W. Kim , K. Keum , H. Lee , G. Jung , M. Park , Y. H. Lee , S. Kim , J. S. Ha , Chem. Eng. J. 2023, 457, 141278.

[35] R. Jin , J. Xu , L. Duan , G. Gao , Carbohydr. Polym. 2021, 268, 118240. 34127222 10.1016/j.carbpol.2021.118240

[36] Y. Zhao , F. Wang , J. Liu , D. Gan , B. Lei , J. Shao , W. Wang , Q. Wang , X. Dong , ACS Appl. Mater. Interfaces 2023, 15, 28664. 37271945 10.1021/acsami.3c05943

[37] X. Du , J. Zhai , X. Li , Y. Zhang , N. Li , X. Xie , ACS Sens. 2021, 6, 1990. 34044533 10.1021/acssensors.1c00756

[38] B. Xue , H. Sheng , Y. Li , L. Li , W. Di , Z. Xu , L. Ma , X. Wang , H. Jiang , M. Qin , Natl. Sci. Rev. 2022, 9, nwab147. 35974839 10.1093/nsr/nwab147 PMC9375542

[39] D. Boateng , X. Li , Y. Zhu , H. Zhang , M. Wu , J. Liu , Y. Kang , H. Zeng , L. Han , Biosens. Bioelectron. 2024, 261, 116499. 38896981 10.1016/j.bios.2024.116499

[40] S. Raschka , J. Patterson , C. Nolet , Information 2020, 11, 193.

[41] Y. H. Jung , S. K. Hong , H. S. Wang , J. H. Han , T. X. Pham , H. Park , J. Kim , S. Kang , C. D. Yoo , K. J. Lee , Adv. Mater. 2020, 32, 1904020. 10.1002/adma.201904020 31617274

[42] X. Xia , X. Cao , B. Zhang , L. Zhang , J. Dong , J. Qin , P. Xuan , L. Liu , Y. Sun , W. Fan , Macromol. Rapid Commun. 2024, 2400379. 10.1002/marc.202400379 38940242

[43] X. Zhong , P. Sun , R. Wei , H. Dong , S. Jiang , J. Mater. Chem. A 2022, 10, 15080.

[44] L. Hu , P. L. Chee , S. Sugiarto , Y. Yu , C. Shi , R. Yan , Z. Yao , X. Shi , J. Zhi , D. Kai , H.‐D. Yu , W. Huang , Adv. Mater. 2023, 35, 2205326. 10.1002/adma.202205326 36037508

[45] N. Dai , I. M. Lei , Z. Li , Y. Li , P. Fang , J. Zhong , Nano Energy 2023, 105, 108041.

[46] X. Xiao , J. Yin , J. Xu , T. Tat , J. Chen , ACS Nano 2024, 18, 22734. 39145724 10.1021/acsnano.4c05851 PMC13046296

[47] H. Niu , F. Yin , E. S. Kim , W. Wang , D. Y. Yoon , C. Wang , J. Liang , Y. Li , N. Y. Kim , InfoMat 2023, 5, 12412.

[48] Y. Qiu , H. Ye , H. Zhang , Y. Zheng , Computer‐Aided Design 2024, 166, 103631.

[49] I. C. Karaoglu , A. O. Kebabci , S. Kizilel , ACS Appl. Mater. Interfaces 2023, 15, 44796. 37704030 10.1021/acsami.3c12207

[50] Y. Ma , D. Zhang , Z. Wang , H. Zhang , H. Xia , R. Mao , H. Cai , H. Luan , ACS Appl. Mater. Interfaces 2023, 15, 29413. 37280727 10.1021/acsami.3c02014

[51] B. Song , X. Dai , X. Fan , H. Gu , J. Mater. Sci. Technol. 2024, 181, 91.

[52] H. Zhang , D. Zhang , Z. Wang , G. Xi , R. Mao , Y. Ma , D. Wang , M. Tang , Z. Xu , H. Luan , ACS Appl. Mater. Interfaces 2023, 15, 5128. 36658100 10.1021/acsami.2c17904

[53] L. Wang , M. Zhou , T. Xu , X. Zhang , Chem. Eng. J. 2022, 433, 134625.

[54] J. Chen , X. Xia , X. Yan , W. Wang , X. Yang , J. Pang , R. Qiu , S. Wu , ACS Appl. Mater. Interfaces 2023, 15, 46440. 37725344 10.1021/acsami.3c06809

[55] F. V. Lavrentev , I. S. Rumyantsev , A. S. Ivanov , V. V. Shilovskikh , O. Y. Orlova , K. G. Nikolaev , D. V. Andreeva , E. V. Skorb , ACS Appl. Mater. Interfaces 2022, 14, 7321. 35080838 10.1021/acsami.1c22470

[56] X. Ma , W. Wang , X. Cui , Y. Li , K. Yang , Z. Huang , H. Zhang , Small 2024, 20, 2402700. 10.1002/smll.202402700 38726773

[57] M. Mohammed , M. B. Khan , E. B. M. Bashier , Machine Learning: Algorithms and Applications, Crc Press, Boca Raton 2016, p. 226.

[58] X. Su , X. Yan , C. L. Tsai , Wiley Interdisc. Rev.: Comput. Statist. 2012, 4, 275.

[59] S. S. Keerthi , S. K. Shevade , C. Bhattacharyya , K. R. K. Murthy , Neural Comput. 2001, 13, 637.

[60] T. Joachims , presented at European Conference on Machine Learning, Berlin/Heidelberg, Germany 1998, pp. 137–142.

[61] F. Jing , M. Li , H.‐J. Zhang , B. Zhang , in 2003 International Conference on Multimedia and Expo. ICME'03. Proceedings (Cat. No. 03TH8698), IEEE, Baltimore, MD, USA 2003.

[62] T. Bellotti , J. Crook , Expert Syst. Appl. 2009, 36, 3302.

[63] R. Chitrakar , H. Chuanhe , in 2012 Third Asian Himalayas International Conference on Internet, IEEE, Kathmundu, Nepal 2012.

[64] J. R. Quinlan , Mach. Learn. 1986, 1, 81.

[65] S. Naganandhini , P. Shanmugavadivu , Proc. Comp. Sci. 2019, 165, 548.

[66] N. Gordini , V. Veglio , in Business Intelligence: Concepts, Methodologies, Tools, and Applications, IGI Global, Hershey, Pennsylvania, USA 2016.

[67] E. Dumitrescu , S. Hué , C. Hurlin , S. Tokpavi , Eur. J. Operat. Res. 2022, 297, 1178.

[68] L. Breiman , Mach. Learn. 2001, 45, 5.

[69] R. Genuer , J.‐M. Poggi , C. Tuleau‐Malot , Patt. Recognit. Lett. 2010, 31, 2225.

[70] R. Primartha , B. A. Tama , presented at 2017 International Conference on Data and Software Engineering (ICoDSE), IEEE, Palembang, Indonesia 2017.

[71] L. Khaidem , S. Saha , S. R. Dey , arXiv 2016, arXiv:1605.00003.

[72] T. Chen , C. Guestrin , presented at Proceedings of the 22nd ACM SIGKDD International Conference on Knowledge Discovery And Data Mining, ACM (Association for Computing Machinery), NY, USA 2016, pp. 785–794.

[73] S. S. Dhaliwal , A.‐A. Nahid , R. Abbas , Information 2018, 9, 149.

[74] K. Budholiya , S. K. Shrivastava , V. Sharma , J. King Saud University‐Comp. Inform. Sci. 2022, 34, 4514.

[75] G. Hamerly , C. Elkan , Adv. Neural Inform. Process. 2003, 16, 281.

[76] H. Kasuga , H. Yamamoto , M. Okamoto , Syst. Comp. Japan 2000, 31, 33.

[77] V. K. Singh , N. Tiwari , S. Garg , presented at 2011 International Conference on Computational Intelligence and Communication Networks, IEEE, Gwalior, India 2011.

[78] N. Arunkumar , M. A. Mohammed , M. K. Abd Ghani , D. A. Ibrahim , E. Abdulhay , G. Ramirez‐Gonzalez , V. H. C. de Albuquerque , Soft Comp. 2019, 23, 9083.

[79] M. Ester , H.‐P. Kriegel , J. Sander , X. Xu , presented at KDD‐96, AAAI Press, München, Germany 1996.

[80] A. Maćkiewicz , W. Ratajczak , Comput. Geosci. 1993, 19, 303.

[81] W. L. Buntine , A. Jakulin , arXiv 2012, 1207.4125.

[82] M. Mudrova , A. Procházka , presented at Proceedings of the MATLAB Technical Computing Conference, MathWorks, Prague 2005.

[83] H. M. Ebied , in 2012 8th International Conference on Informatics and Systems (INFOS), Cairo, Egypt 2012.

[84] A. Hyvärinen , E. Oja , Neural Networks 2000, 13, 411. 10946390 10.1016/s0893-6080(00)00026-5

[85] S. Makino , S. Araki , R. Mukai , H. Sawada , presented at 2004 IEEE International Symposium on Circuits and Systems (IEEE Cat. No. 04CH37512), IEEE, Vancouver, BC, Canada 2004.

[86] Z. Li , F. Liu , W. Yang , S. Peng , J. Zhou , IEEE Transact. Neural Network. Learn. Syst. 2021, 33, 6999. 10.1109/TNNLS.2021.3084827 34111009

[87] U. Erdenebayar , H. Kim , J.‐U. Park , D. Kang , K.‐J. Lee , J. Korean Med. Sci. 2019, 34. 10.3346/jkms.2019.34.e64 PMC6384436 30804732

[88] D. Han , J. Chen , J. Sun , Int. J. Distribut. Sens. Networks 2019, 15, 155014771983279.

[89] Q. Zhang , D. Zhou , X. Zeng , IEEE Access 2017, 5, 11805.

[90] O. Abdeljaber , S. Sassi , O. Avci , S. Kiranyaz , A. A. Ibrahim , M. Gabbouj , IEEE Transact. Industr. Electron. 2018, 66, 8136.

[91] Q. Li , W. Cai , X. Wang , Y. Zhou , D. D. Feng , M. Chen , presented at 2014 13th International Conference on Control Automation Robotics & Vision (ICARCV), IEEE, Singapore 2014.

[92] B. Kayalibay , G. Jensen , P. van der Smagt , arXiv 2017, 1701.03056.

[93] M. Coşkun , A. Uçar , Ö. Yildirim , Y. Demir , presented at 2017 International Conference on Modern Electrical and Energy Systems (MEES), IEEE, Kremenchuk, Ukraine 2017.

[94] A. Stergiou , R. Poppe , presented at 2019 18th IEEE International Conference on Machine Learning and Applications (ICMLA), IEEE, Boca Raton, FL, USA 2019.

[95] F. Pastor , J. M. Gandarias , A. J. García‐Cerezo , J. M. Gómez‐de‐Gabriel , Sensors 2019, 19, 5356. 31817320 10.3390/s19245356 PMC6960774

[96] L. R. Medsker , L. Jain , Design Appl. 2001, 5, 2.

[97] K. M. Tarwani , S. Edem , Int. J. Eng. Trends Technol 2017, 48, 301.

[98] A. Sherstinsky , Phys. D: Nonlinear Phenomena 2020, 404, 132306.

[99] D. Jyotishi , S. Dandapat , IEEE Sens. J. 2021, 22, 6052.

[100] A. Sarvari , K. Sridevi , Biomed. Signal Process. Control 2023, 83, 104637. 36776947 10.1016/j.bspc.2023.104637 PMC9904992

[101] H. Chen , M. Du , Y. Zhang , C. Yang , Comput. Intel. Neurosci. 2022, 2022, 8431912. 10.1155/2022/8431912 PMC9020897 35463275

[102] F. Huang , X. Li , C. Yuan , S. Zhang , J. Zhang , S. Qiao , IEEE Transact. Neural Network. Learn. Syst. 2021, 33, 4332. 10.1109/TNNLS.2021.3056664 33600326

[103] J. Sangeetha , U. Kumaran , Measurement: Sens. 2023, 25, 100619.

[104] Y. LeCun , Y. Bengio , G. Hinton , Deep Learn. Nat. 2015, 521, 436. 10.1038/nature14539 26017442

[105] A. Shrestha , A. Mahmood , IEEE Access 2019, 7, 53040.

[106] K. Fang , Y. Wan , J. Wei , T. Chen , Langmuir 2023, 39, 16975. 37994525 10.1021/acs.langmuir.3c02444

[107] W. He , X. Ming , Y. Xiang , C. Zhang , H. Zhu , Q. Zhang , S. Zhu , ACS Appl. Mater. Interfaces 2022, 14, 20132. 35470664 10.1021/acsami.2c04510

[108] X. Sun , S. Agate , K. S. Salem , L. Lucia , L. Pal , ACS Appl. Bio Mater. 2021, 4, 140. 10.1021/acsabm.0c01011 35014280

[109] Z. Fu , H. Liu , Q. Lyu , J. Dai , C. Ji , Y. Tian , Chem. Eng. J. 2024, 481, 148526.

[110] J. Sun , Y. Yuan , G. Lu , L. Li , X. Zhu , J. Nie , J. Mater. Chem. C 2019, 7, 11244.

[111] A. Barhoum , O. Sadak , I. A. Ramirez , N. Iverson , Adv. Colloid Interface Sci. 2023, 317, 102920. 37207377 10.1016/j.cis.2023.102920

[112] W. Li , L. Li , Z. Liu , S. Zheng , Q. Li , F. Yan , Adv. Mater. 2023, 35, 2301383. 10.1002/adma.202301383 37094299

[113] M. Wang , J. Hu , M. D. Dickey , JACS Au 2022, 2, 2645. 36590265 10.1021/jacsau.2c00489 PMC9795568

[114] M. Wang , J. Hu , M. D. Dickey , NPG Asia Mater. 2023, 15, 66.

[115] L. Zhao , B. Wang , Z. Mao , X. Sui , X. Feng , Chem. Eng. J. 2022, 433, 133500.

[116] E. M. Ahmed , J. Adv. Res. 2015, 6, 105.25750745

[117] F. Yokoyama , I. Masada , K. Shimamura , T. Ikawa , K. Monobe , Colloid Polym. Sci. 1986, 264, 595.

[118] U. Gulyuz , O. Okay , Macromolecules 2014, 47, 6889.

[119] X. Xue , Y. Hu , S. Wang , X. Chen , Y. Jiang , J. Su , Bioact. Mater. 2022, 12, 327. 35128180 10.1016/j.bioactmat.2021.10.029 PMC8784310

[120] G. Sun , P. Wang , Y. Jiang , H. Sun , C. Meng , S. Guo , Soft Sci. 2022, 2, 17.

[121] W. Hu , Z. Wang , Y. Xiao , S. Zhang , J. Wang , Biomater. Sci. 2019, 7, 843. 30648168 10.1039/c8bm01246f

[122] W. E. Hennink , C. F. van Nostrum , Adv. Drug Delivery Rev. 2012, 64, 223. 10.1016/s0169-409x(01)00240-x 11755704

[123] N. Reddy , R. Reddy , Q. Jiang , Trends Biotechnol. 2015, 33, 362. 25887334 10.1016/j.tibtech.2015.03.008

[124] A. Oryan , A. Kamali , A. Moshiri , H. Baharvand , H. Daemi , Int. J. Biol. Macromol. 2018, 107, 678. 28919526 10.1016/j.ijbiomac.2017.08.184

[125] Y. Liu , F. Zhuo , J. Zhou , L. Kuang , K. Tan , H. Lu , J. Cai , Y. Guo , R. Cao , Y. Fu , ACS Appl. Mater. Interfaces 2022, 14, 54276. 36417548 10.1021/acsami.2c17943

[126] H. Wang , R. Shang , J. Chen , X. Jin , K. Chen , B. Huang , H. Chen , Q.‐L. Lu , Nano Energy 2024, 128, 109843.

[127] L. Zhao , H. Xu , L. Liu , Y. Zheng , W. Han , L. Wang , Adv. Sci. 2023, 10, 2303922. 10.1002/advs.202303922 PMC10602575 37672883

[128] H. Ma , H. Qin , X. Xiao , N. Liu , S. Wang , J. Li , S. Shen , S. Dai , M. Sun , P. Li , X. Pan , M. Huang , B. Lu , J. Chen , L. Wu , InfoMat 2023, 5, 12419.

[129] J. Zhou , Y. Liu , F. Zhuo , H. Chen , H. Cao , Y. Fu , J. Xie , H. Duan , Chem. Eng. J. 2024, 479, 147790.

[130] Z. Wang , M. Bu , K. Xiu , J. Sun , N. Hu , L. Zhao , L. Gao , F. Kong , H. Zhu , J. Song , D. Lau , A. Flexible , Nano Energy 2022, 107978.

[131] Y. Xin , W. Gao , G. Zeng , S. Chen , J. Shi , W. Wang , K. Ma , B. Qu , J. Fu , X. He , Int. J. Biol. Macromol. 2024, 258, 129068. 38158069 10.1016/j.ijbiomac.2023.129068

[132] G. Zhu , N. Javanmardia , L. Qian , F. Jin , T. Li , S. Zhang , Y. He , Y. Wang , X. Xu , T. Wang , Z.‐Q. Feng , Int. J. Biol. Macromol. 2024, 281, 136115. 39349076 10.1016/j.ijbiomac.2024.136115

[133] C.‐C. Yan , W. Li , Z. Liu , S. Zheng , Y. Hu , Y. Zhou , J. Guo , X. Ou , Q. Li , J. Yu , L. Li , M. Yang , Q. Liu , F. Yan , Adv. Funct. Mater. 2024, 34, 2314408.

[134] M. Salsamendi , L. Rubatat , D. Mecerreyes , in Electrochemistry in Ionic Liquids: Volume 1: Fundamentals, (Ed.: A. A. J. Torriero ), Springer International Publishing, Cham 2015.

[135] J. Xie , X. Li , Z. He , L. Fan , D. Yao , Y. Zheng , Mater. Horiz. 2024, 11, 238. 37909216 10.1039/d3mh01587d

[136] E. Andrzejewska , A. Marcinkowska , A. Zgrzeba , Polimery 2017, 62, 344.

[137] A. K. Tripathi , Mater. Today Energy 2021, 20, 100643.

[138] M. Wu , M. Pan , C. Qiao , Y. Ma , B. Yan , W. Yang , Q. Peng , L. Han , H. Zeng , Chem. Eng. J. 2022, 450, 138212.

[139] F. Li , J. Han , T. Cao , W. Lam , B. Fan , W. Tang , S. Chen , K. L. Fok , L. Li , Proc. Natl. Acad. Sci. USA 2019, 116, 11259. 31110004 10.1073/pnas.1903376116 PMC6561259

[140] W. Li , Y. Wen , K. Wang , Z. Ding , L. Wang , Q. Chen , L. Xie , H. Xu , H. Zhao , Nat. Commun. 2024, 15, 2603. 38521777 10.1038/s41467-024-46866-9 PMC10960799

[141] T. Xu , J. Wang , S. Zhao , D. Chen , H. Zhang , Y. Fang , N. Kong , Z. Zhou , W. Li , H. Wang , Nat. Commun. 2023, 14, 3880. 37391398 10.1038/s41467-023-39648-2 PMC10313671

[142] L. Garcia‐del Rio , P. Diaz‐Rodriguez , M. Landin , Mater. Sci. Eng., C 2020, 106, 110252. 10.1016/j.msec.2019.110252 31753360

[143] S. Zheng , H. You , K. Lam , H. Li , Adv. Theory Simul. 2024, 7, 2300776.

[144] Y. Wang , T. Wallmersperger , A. Ehrenhofer , Eng. Rep. 2024, 6, 12893.

[145] S. Xu , X. Chen , S. Wang , Z. Chen , P. Pan , Q. Huang , Regenerat. Biomater. 2024, 11, rbae109. 10.1093/rb/rbae109 PMC11422183 39323746

[146] M. Seifermann , P. Reiser , P. Friederich , P. A. Levkin , Small Methods 2023, 7, 2300553. 10.1002/smtd.202300553 37287430

[147] F. Xu , B. Corbett , S. Bell , C. Zhang , M. Budi Hartono , Z. J. Farsangi , J. MacGregor , T. Hoare , Biomacromolecules 2019, 21, 214. 31686502 10.1021/acs.biomac.9b01132

[148] W. Zhang , X. Zhang , W. Zhao , X. Wang , ACS Appl. Polym. Mater 2023, 5, 2628.

[149] H. Zhang , D. Zhang , H. Luan , Z. Wang , P. Zhang , G. Xi , X. Ji , Langmuir 2023, 39, 16199. 37906584 10.1021/acs.langmuir.3c02666

[150] C. Yang , D. Zhang , D. Wang , H. Luan , X. Chen , W. Yan , ACS Appl. Mater. Interfaces 2023, 15, 5811. 36648277 10.1021/acsami.2c18989

[151] F. Wang , D. Song , C. Zhou , X. Li , Y. Huang , W. Xu , G. Liu , S. Zhou , ACS Appl. Mater. Interfaces 2024, 16, 41583. 39046871 10.1021/acsami.4c10043

[152] K. Li , D. Zhang , H. Zhang , D. Wang , Z. Xu , H. Cai , H. Xia , ACS Appl. Mater. Interfaces 2023, 15, 32993. 37381708 10.1021/acsami.3c06597

[153] N. Li , Z. Wang , X. Yang , Z. Zhang , W. Zhang , S. Sang , H. Zhang , Adv. Funct. Mater. 2024, 34, 2314419.

[154] J. Huang , J. Gu , J. Liu , J. Guo , H. Liu , K. Hou , X. Jiang , X. Yang , L. Guan , J. Mater. Chem. A 2021, 9, 16345.

[155] H. Wu , H. Qi , X. Wang , Y. Qiu , K. Shi , H. Zhang , Z. Zhang , W. Zhang , Y. Tian , J. Mater. Chem. C 2022, 10, 8206.

[156] R. Wan , J. Yu , Z. Quan , H. Ma , J. Li , F. Tian , W. Wang , Y. Sun , J. Liu , D. Gao , Chem. Eng. J. 2024, 490, 151454.

[157] H. Jiang , C. Ou , D. Zhang , X. Hu , Y. Ma , M. Wang , Y. Huang , L. Xiao , ACS Appl. Polym. Mater 2023, 5, 6828.

[158] W. Li , S. Wu , S. Li , X. Zhong , X. Zhang , H. Qiao , M. Kang , J. Chen , P. Wang , L.‐Q. Tao , ACS Appl. Mater. Interfaces 2023, 15, 45106. 37699573 10.1021/acsami.3c08709

[159] W. Li , S. Wu , M. Kang , X. Zhang , X. Zhong , H. Qiao , J. Chen , P. Wang , L. Tao , J. Mater. Sci. Technol. 2024, 201, 130.

[160] Y. Sun , J. Huang , Y. Cheng , J. Zhang , Y. Shi , L. Pan , SmartMat 2024, 5, 1269.

[161] Z. Zhang , M. Sang , Y. Pan , Z. Li , S. Liu , J. Wu , X. Wang , S. Duan , X. Gong , Chem. Eng. J. 2024, 496, 154227.

[162] Y. Zhang , Z. Li , Z. Xu , M. Xiao , Y. Yuan , X. Jia , R. Shi , L. Zhang , P. Wan , Aggregate 2024, 5, 566.

[163] L. You , Z. Zheng , W. Xu , Y. Wang , W. Xiong , C. Xiong , S. Wang , Int. J. Biol. Macromol. 2024, 263, 130439. 38423420 10.1016/j.ijbiomac.2024.130439

[164] H. Wang , Q. Ding , Y. Luo , Z. Wu , J. Yu , H. Chen , Y. Zhou , H. Zhang , K. Tao , X. Chen , J. Fu , J. Wu , Adv. Mater. 2024, 36, 2309868. 10.1002/adma.202309868 38095146

[165] Q. Li , X. Zhi , Y. Xia , S. Han , W. Guo , M. Li , X. Wang , ACS Appl. Mater. Interfaces 2023, 15, 19435. 37035900 10.1021/acsami.3c00432

[166] J. Shi , Y. Dai , Y. Cheng , S. Xie , G. Li , Y. Liu , J. Wang , R. Zhang , N. Bai , M. Cai , Sci. Adv. 2023, 9, adf8831. 10.1126/sciadv.adf8831 PMC9984179 36867698

[167] D. Lv , X. Li , X. Huang , C. Cao , L. Ai , X. Wang , S. K. Ravi , X. Yao , Adv. Mater. 2024, 36, 2309821. 10.1002/adma.202309821 37993105

[168] Z. Sun , S. Wang , Y. Zhao , Z. Zhong , L. Zuo , Adv. Intel. Syst. 2022, 4, 2200089.

[169] K. Tao , J. Yu , J. Zhang , A. Bao , H. Hu , T. Ye , Q. Ding , Y. Wang , H. Lin , J. Wu , ACS Nano 2023, 17, 16160. 37523784 10.1021/acsnano.3c05253

[170] K. K. Kim , I. Ha , M. Kim , J. Choi , P. Won , S. Jo , S. H. Ko , Nat. Commun. 2020, 11, 2149. 32358525 10.1038/s41467-020-16040-y PMC7195472

[171] K. K. Kim , M. Kim , K. Pyun , J. Kim , J. Min , S. Koh , S. E. Root , J. Kim , B.‐N. T. Nguyen , Y. Nishio , S. Han , J. Choi , C. Y. Kim , J. B. H. Tok , S. Jo , S. H. Ko , Z. Bao , Nat. Electron. 2023, 6, 64.

[172] Z. Miao , H. Tan , L. Gustavsson , Y. Zhou , Q. Xu , O. Ikkala , B. Peng , Small 2024, 20, 2305195. 10.1002/smll.202305195 37803472

[173] S. Zheng , X. Chen , K. Shen , Y. Cheng , L. Ma , X. Ming , ACS Appl. Mater. Interfaces 2024, 16, 4035. 38200632 10.1021/acsami.3c16195

[174] H. Jiang , S. Jiang , G. Chen , Y. Lan , Adv. Funct. Mater. 2024, 34, 2307313.

[175] J. W. Suen , N. K. Elumalai , S. Debnath , N. M. Mubarak , C. I. Lim , M. M. Reddy , Adv. Mater. Interfaces 2022, 9, 2201405.

[176] Y. Yang , Y. An , Z. Yang , B. Fu , Z. Chen , X. Zheng , B. Xu , W. Shen , Y. Wang , Y. He , ACS Appl. Mater. Interfaces 2023, 15, 23749. 37143329 10.1021/acsami.3c02629

[177] Y. Song , T. H. Nguyen , D. Lee , J. Kim , ACS Appl. Mater. Interfaces 2024, 16, 9551. 38331574 10.1021/acsami.3c18588

[178] X. Zhong , T. Song , H. Dong , S. Jiang , R. Wei , Chem. Eng. J. 2023, 474, 145686.

[179] R. Guo , Y. Fang , Z. Wang , A. Libanori , X. Xiao , D. Wan , X. Cui , S. Sang , W. Zhang , H. Zhang , J. Chen , Adv. Funct. Mater. 2022, 32, 2204803.

[180] F. Luo , B. Chen , X. Ran , W. Ouyang , Y. Yao , L. Shang , Nano Energy 2023, 118, 109035.

[181] S. Hao , T. Li , X. Yang , H. Song , Ultrastretchable, A. , ACS Appl. Mater. Interfaces 2022, 14, 2029. 34958556 10.1021/acsami.1c21325

[182] J. Zhang , E. Liu , S. Hao , X. Yang , T. Li , C. Lou , M. Run , H. Song , Chem. Eng. J. 2022, 431, 133949.

[183] X. Wang , Y. Xiao , F. Deng , Y. Chen , H. Zhang , Biosensors 2021, 22, 11. 10.3390/bios11060198 PMC8234407 34208524

[184] J. Hua , M. Su , X. Sun , J. Li , Y. Sun , H. Qiu , Y. Shi , L. Pan , Biosensors 2023, 13, 696. 37504095 10.3390/bios13070696 PMC10377104

[185] S. Baik , J. Lee , E. J. Jeon , B.‐y. Park , D. W. Kim , J. H. Song , H. J. Lee , S. Y. Han , S.‐W. Cho , C. Pang , Sci. Adv. 2021, 7, abf5695. 10.1126/sciadv.abf5695 PMC8208721 34134988

[186] S. Kalasin , P. Sangnuang , W. Surareungchai , Anal. Chem. 2022, 94, 6842. 35467846 10.1021/acs.analchem.2c00782

[187] Y. Xi , S. Cheng , S. Chao , Y. Hu , M. Cai , Y. Zou , Z. Liu , W. Hua , P. Tan , Y. Fan , Z. Li , Nano Res. 2023, 16, 11674.

[188] L. Wang , T. Xu , X. He , X. Zhang , J. Mater. Chem. C 2021, 9, 14938.

[189] L. Wang , T. Xu , C. Fan , X. Zhang , IScience 2021, 24, 102028. 33490926 10.1016/j.isci.2020.102028 PMC7809499

[190] J. Zhang , Z. Liu , Y. Tang , S. Wang , J. Meng , F. Li , Anal. Chem. 2024, 96, 1205. 38191284 10.1021/acs.analchem.3c04368

[191] Y. Song , R. Y. Tay , J. Li , C. Xu , J. Min , E. Shirzaei Sani , G. Kim , W. Heng , I. Kim , W. Gao , Sci. Adv. 2023, 9, adi6492. 10.1126/sciadv.adi6492 PMC10499321 37703361

[192] H. Xu , Y. Wang , S. Lin , W. Deng , D. Peng , Q. Cui , Y. Xue , Genom. Proteom. Bioinform. 2018, 16, 244. 10.1016/j.gpb.2018.06.004 PMC6205080 30244175

[193] J. Qin , J. Guo , G. Tang , L. Li , S. Q. Yao , Angew. Chem., Int. Ed. 2023, 62, 202218412. 10.1002/anie.202218412 36815677

[194] D. Won , H. Kim , J. Kim , H. Kim , M. W. Kim , J. Ahn , K. Min , Y. Lee , S. Hong , J. Choi , C. Y. Kim , T.‐S. Kim , S. H. Ko , Nat. Electron. 2024, 7, 475.

[195] D. Won , J. Kim , J. Choi , H. Kim , S. Han , I. Ha , J. Bang , K. K. Kim , Y. Lee , T.‐S. Kim , J.‐H. Park , C. Y. Kim , S. H. Ko , Sci. Adv. 2022, 8, abo3209. 10.1126/sciadv.abo3209 PMC9177068 35675404

[196] D. Won , H. Kim , T.‐S. Kim , S. H. Ko , Appl. Phys. Rev. 2025, 12, 031302.

[197] J. Kim , D. Won , T. H. Kim , C. Y. Kim , S. H. Ko , Biosens. Bioelectron. 2024, 258, 116327. 38703496 10.1016/j.bios.2024.116327

[198] J. Zhou , T. Chen , Z. He , L. Sheng , X. Lu , J. Mater. Chem. C 2023, 11, 13476.

[199] G. Zhong , Q. Liu , Y. Huang , H. Geng , T. Xu , A. Wideband , Adv. Mater. 2025, 08206. 10.1002/adma.202508206 40838529

[200] H. Xu , W. Zheng , Y. Zhang , D. Zhao , L. Wang , Y. Zhao , W. Wang , Y. Yuan , J. Zhang , Z. Huo , Y. Wang , N. Zhao , Y. Qin , K. Liu , R. Xi , G. Chen , H. Zhang , C. Tang , J. Yan , Q. Ge , H. Cheng , Y. Lu , L. Gao , Nat. Commun. 2023, 14, 7769. 38012169 10.1038/s41467-023-43664-7 PMC10682047

[201] Z. Lu , M. Chen , T. Liu , C. Wu , M. Sun , G. Su , X. Wang , Y. Wang , H. Yin , X. Zhou , J. Ye , Y. Shen , H. Rao , ACS Appl. Mater. Interfaces 2023, 15, 9800. 36750421 10.1021/acsami.2c16565

[202] A. Jayakumar , V. K. Jose , J.‐M. Lee , Small Methods 2020, 4, 1900735.

[203] X. Lin , H. Yan , L. Zhao , N. Duan , Z. Wang , S. Wu , Crit. Rev. Food Sci. Nutr. 2024, 64, 6395. 36660935 10.1080/10408398.2023.2168619

[204] F. Cui , S. Zheng , D. Wang , L. Ren , T. Wang , Y. Meng , R. Ma , S. Wang , X. Li , T. Li , J. Li , Int. J. Biol. Macromol. 2024, 259, 129258. 38218291 10.1016/j.ijbiomac.2024.129258

[205] Z. Li , P. Song , G. Li , Y. Han , X. Ren , L. Bai , J. Su , Mater. Today Bio. 2024, 25, 101014. 10.1016/j.mtbio.2024.101014 PMC10924066 38464497

[206] X. Fan , S. Liu , Z. Jia , J. J. Koh , J. C. C. Yeo , C.‐G. Wang , N. E. Surat'Man , X. J. Loh , J. L. Bideau , C. He , Chem. Soc. Rev. 2023, 52, 2497. 36928878 10.1039/d2cs00652a

[207] M. Oudah , A. Al‐Naji , J. Chahl , J. Imag. 2020, 6, 73. 10.3390/jimaging6080073 PMC8321080 34460688

